# Supplementary material for: A flexible artificial intrinsic-synaptic tactile sensory organ
Source: Nat Commun. 2020 Jun 2;11:2753. doi: 10.1038/s41467-020-16606-w (PMC7265430; doi:10.1038/s41467-020-16606-w)
Supplement: Supplementary file 1 — Supplementary Information [file 41467_2020_16606_MOESM1_ESM.pdf]

**Supplementary Information**

# A Flexible Artificial Intrinsic-Synaptic Tactile Sensory Organ

**Lee et al.**

**Supplementary Figures 1~24**

**Supplementary Table 1**

**Supplementary Notes 1~ 10**

## Supplementary Figures

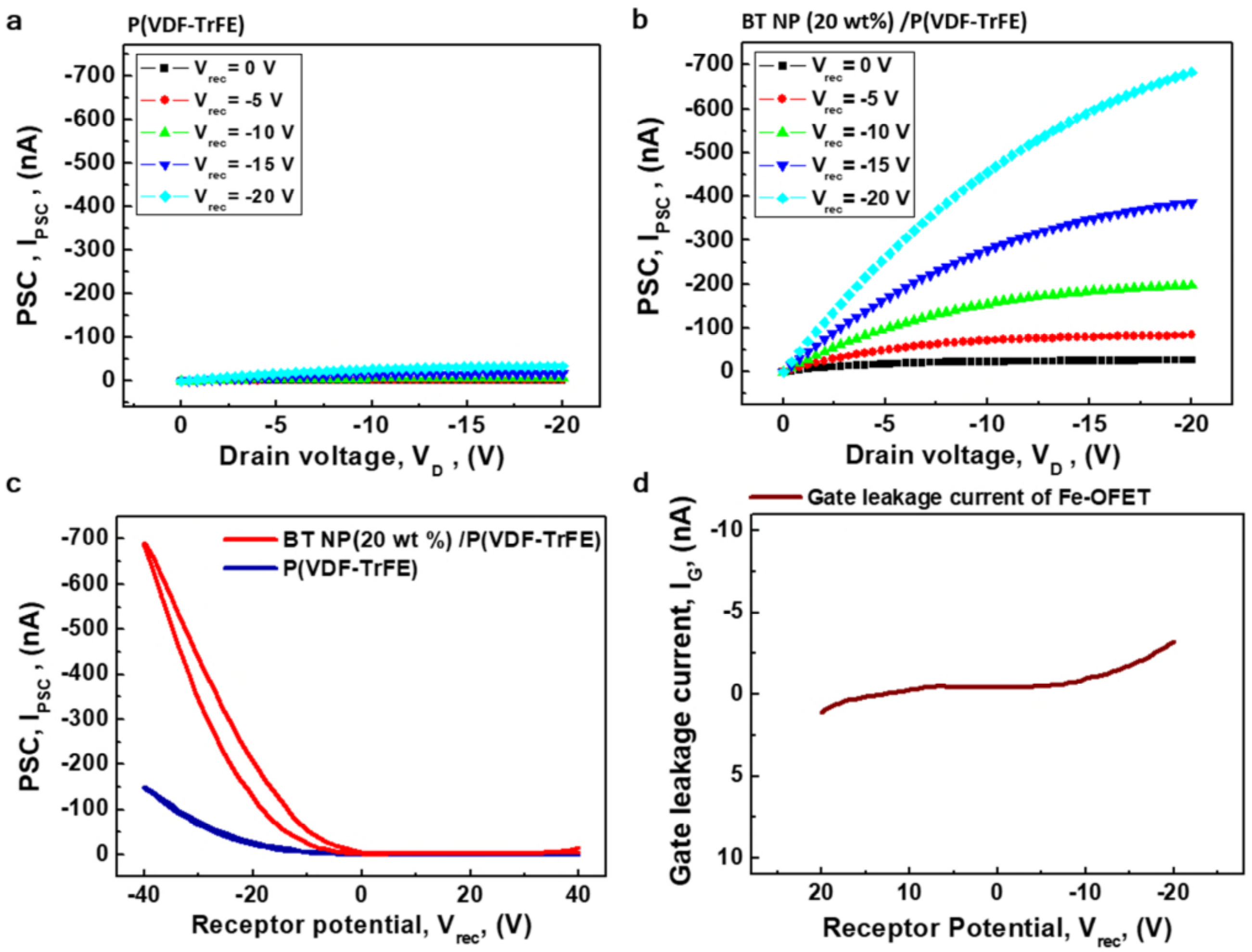


**Supplementary Figure 1. Basic characteristics of Fe-OFET and P-E curve of ferroelectric film. a**, Output characteristics of Fe-OFET with P(VDF-TrFE) gate insulating layer. **b**, Output characteristics of Fe-OFET with BT NP(20wt%)/P(VDF-TrFE) nanocomposite gate insulating layer. **c**, Transfer curves of Fe-OFET with BT NP(20wt%)/P(VDF-TrFE) and P(VDF-TrFE). $I_{PSC}$ and hysteresis are higher for Fe-OFET with BT NP(20wt%)/P(VDF-TrFE) compared to that with P(VDF-TrFE) only due to higher dielectric constant and lower coercive field. **d**, Gate leakage current of Fe-OFET with BT NP(20wt%) /P(VDF-TrFE) gate insulating layer.

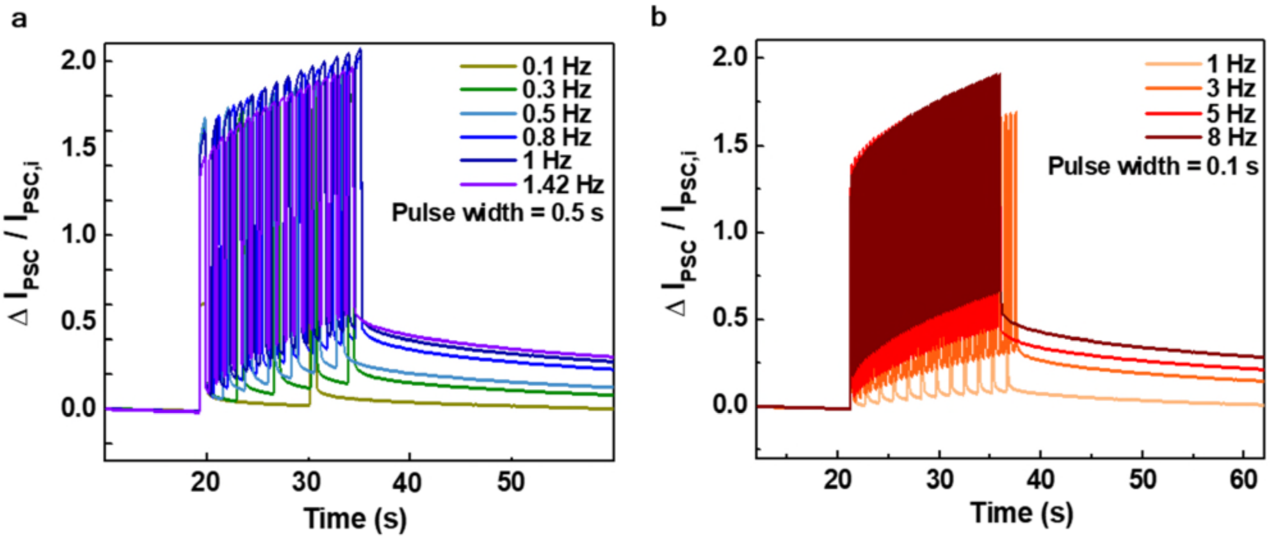


**Supplementary Figure 2. Changes in $I_{PSC}$ and $\Delta I_{PSC}/I_{PSC,i}$ with different frequency range. a**, Changes in $I_{PSC}$ and $\Delta I_{PSC}/I_{PSC,i}$ in response to stimulation with frequencies ranging from 0.1 Hz to 1.42 Hz with a pulse width of 0.5 s. Here, $I_{PSC,i}$ indicates the initial value of the PSC. **b**, Changes in $I_{PSC}$ and $\Delta I_{PSC}/I_{PSC,i}$ according to receptor potential frequencies of 1 - 8 Hz with a pulse width of 0.5 s.

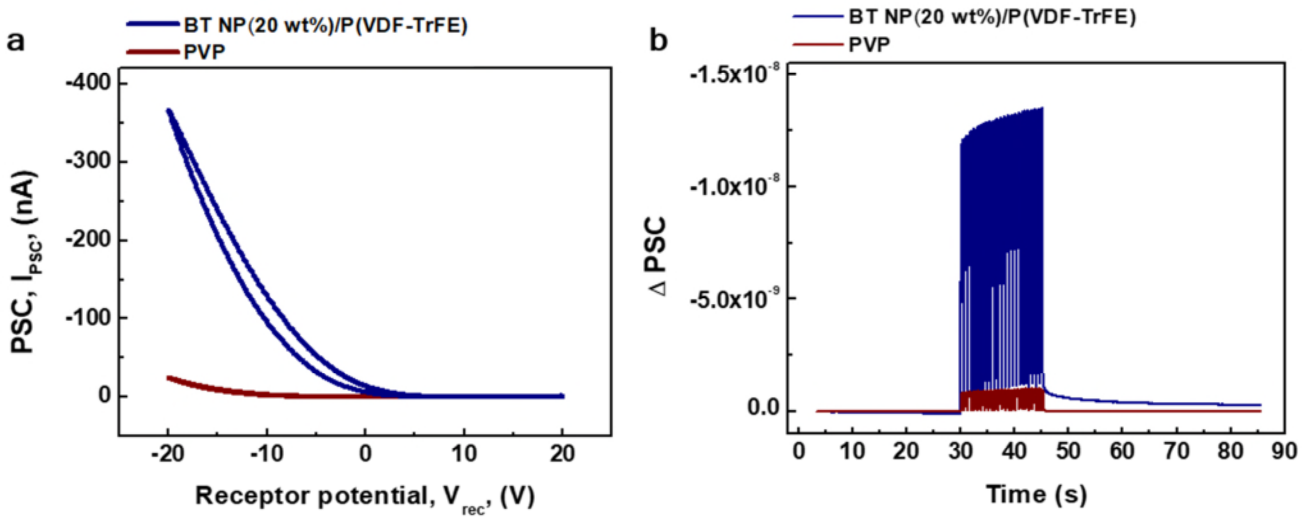


**Supplementary Figure 3. Comparison of characteristics OFET with ferroelectric and non-ferroelectric gate dielectric. a,** Transfer curves of OFET with non-ferroelectric PVP (polyvinylpyrrolidone) gate dielectric and Fe-OFET with the ferroelectric nanocomposite gate dielectric (BT NP(20 wt%)/P(VDF-TrFE)). **b**, Change of PSC (ΔPSC) in OFET with non-ferroelectric PVP gate dielectric and Fe-OFET with the ferroelectric nanocomposite gate dielectric (BT NP(20 wt%)/P(VDF-TrFE)) when the 1.42 Hz of pulses are applied on gate electrode (pulse width = 0.5 s, amplitude = -10 V).

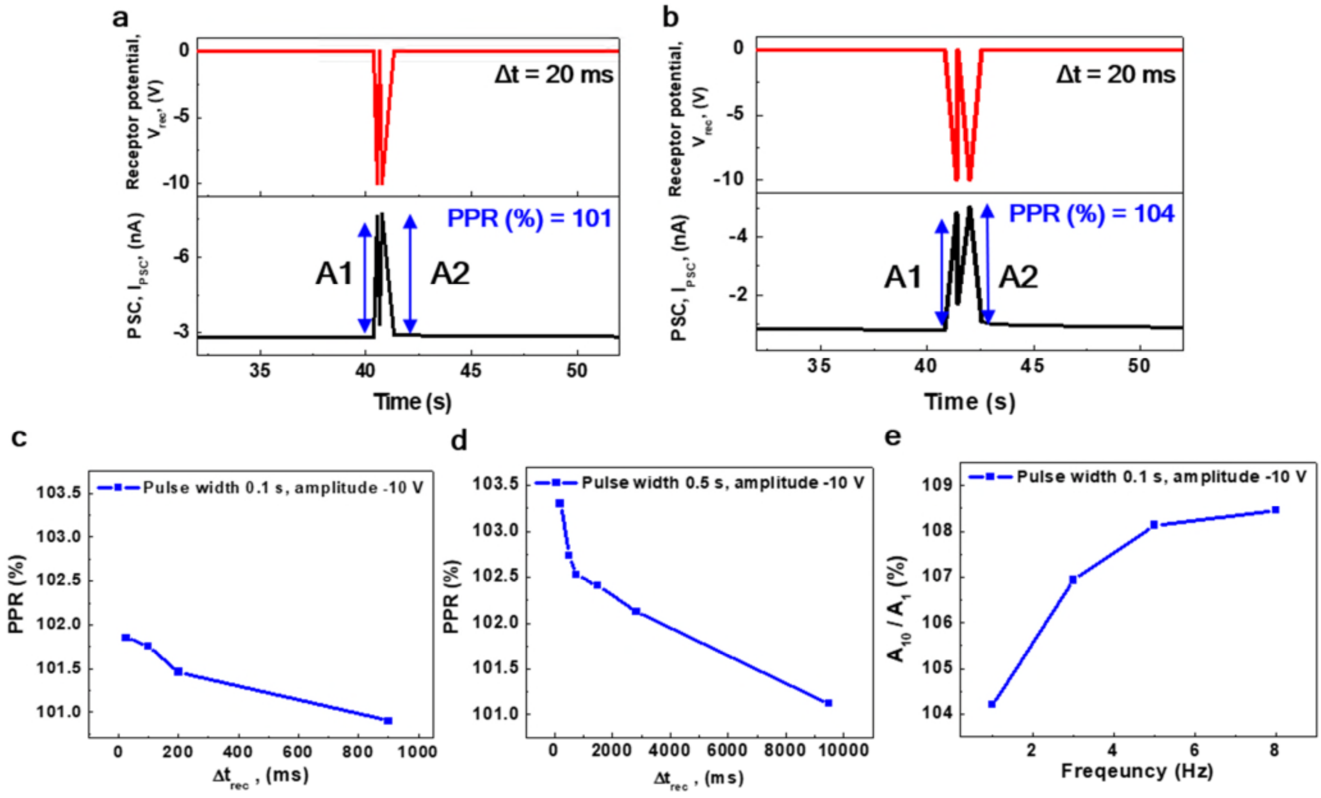


**Supplementary Figure 4. Paired pulse ratio (PPR).** When two identical pulses with a pulse width of **a**, 0.1 s and **b**, 0.5 s were applied continuously, the second PSC peak current increased. PPR values according to the time interval ($\Delta t_{rec}$) between two pulses with a pulse width of **c**, 0.1 s or **d**, 0.5 s. **e**, Ratio of the first peak of PSC ($A_1$) to the tenth peak of PSC ($A_{10}$) according to the frequency of the pulse when successive pulses (pulse width of 0.1 s) were applied.

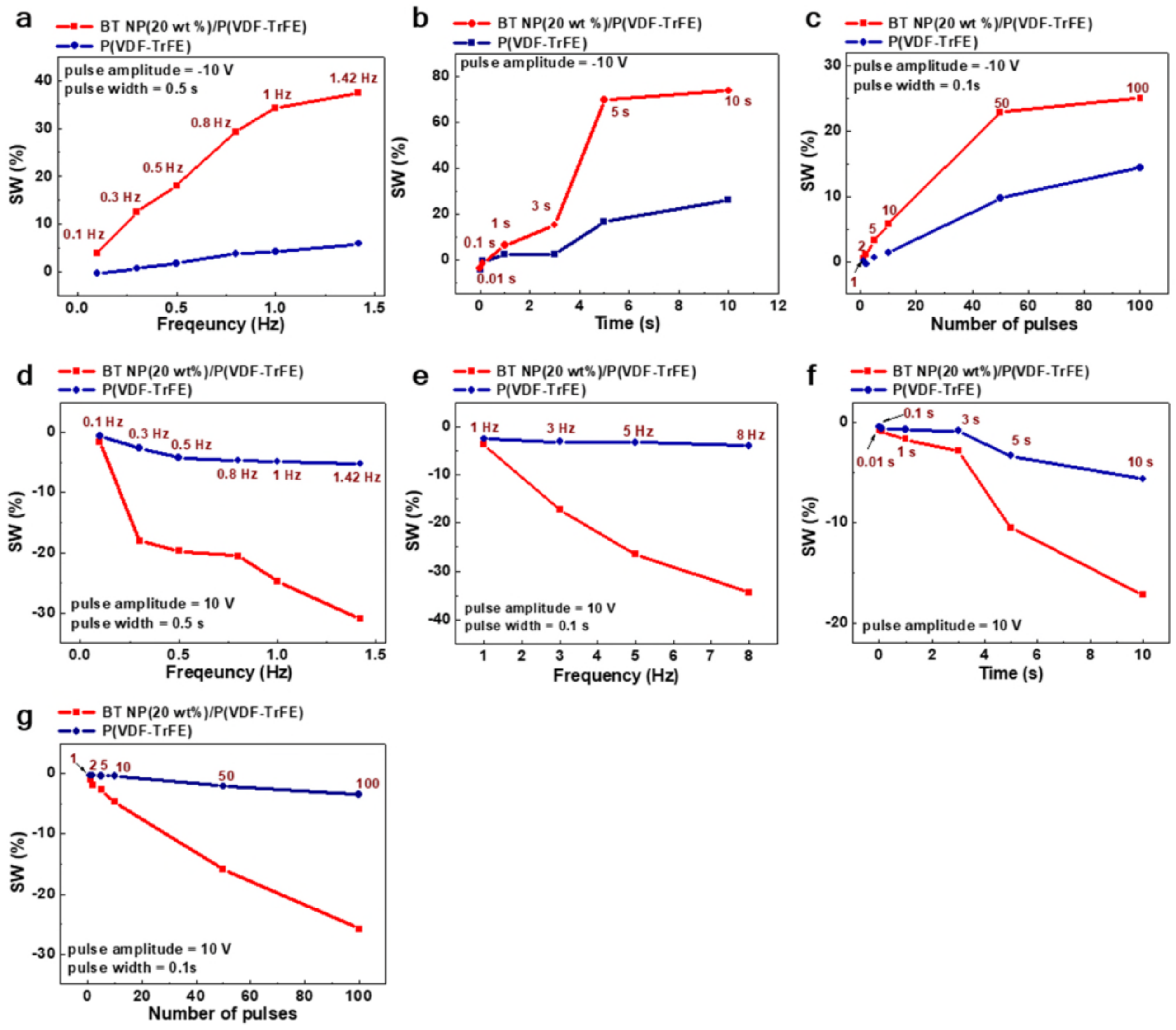


**Supplementary Figure 5. Analysis of synaptic weights.** Synaptic weight (SW) values of devices with BT NP(20 wt%)/P(VDF-TrFE) (red) or P(VDF-TrFE) (blue) for **a**, different frequencies of $V_{rec}$ (pulse width of 0.5 s and amplitude of -10 V), **b**, different durations of $V_{rec}$ (amplitude of -10 V), **c**, different number of pulses of $V_{rec}$ (pulse width of 0.1 s and amplitude of -10 V), **d**, different frequencies of $V_{rec}$ (pulse width of 0.5 s and amplitude of 10 V), **e**, different frequencies of pulses of $V_{rec}$ (pulse width of 0.1 s and amplitude of 10 V), **f**, different pulse durations of $V_{rec}$ (amplitude of 10 V) and **g**, different number of pulses of $V_{rec}$ (pulse width of 0.1 s and amplitude of 10 V).

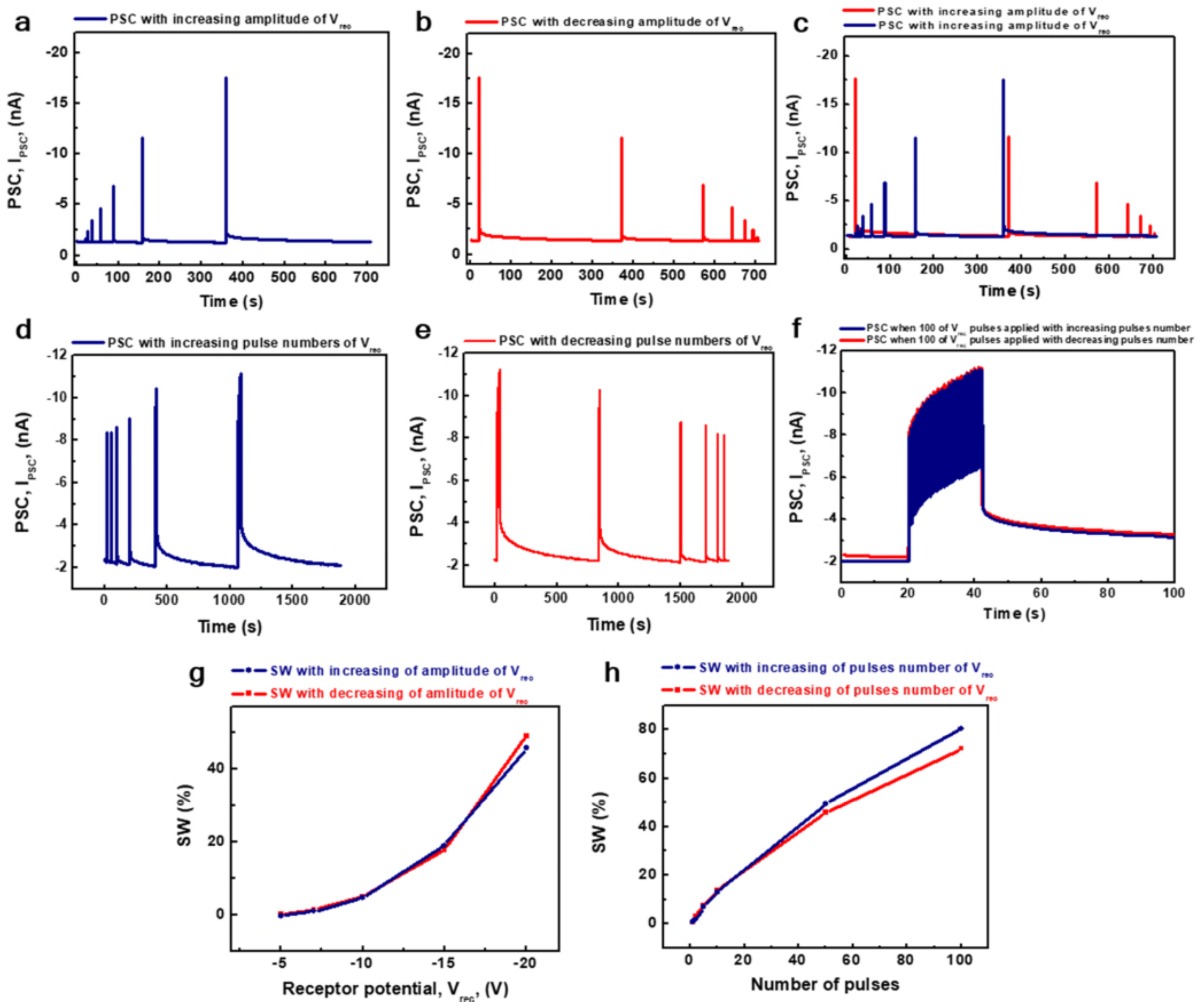


**Supplementary Figure 6. Repeatability test with the full recovery of Fe-OFET. a**, PSC when applying the increasing amplitude of $V_{rec}$ from -1V to -20V with the full recovery of current. **b**, PSC when decreasing amplitude of $V_{rec}$ from -20V to -1V with the full recovery of current. **c**, Merged PSC to demonstrate the repeatability of Fe-OFET. c, Compare the PSC with increasing and decreasing amplitude of $V_{rec}$. **d**, PSC when applying the increasing pulses number of Vrec from 1 to 100. **e**, PSC when applying the decreasing pulses number of $V_{rec}$ from 100 to 1. **f**, Compare the PSC at 100 number of pulses applied in case of applying increasing and decreasing number of $V_{rec}$ pulses. **g**, SW with increasing and decreasing of amplitude of $V_{rec}$ with the full recovery. **h**, SW with increasing and decreasing of pulses number of $V_{rec}$ with the full recovery.

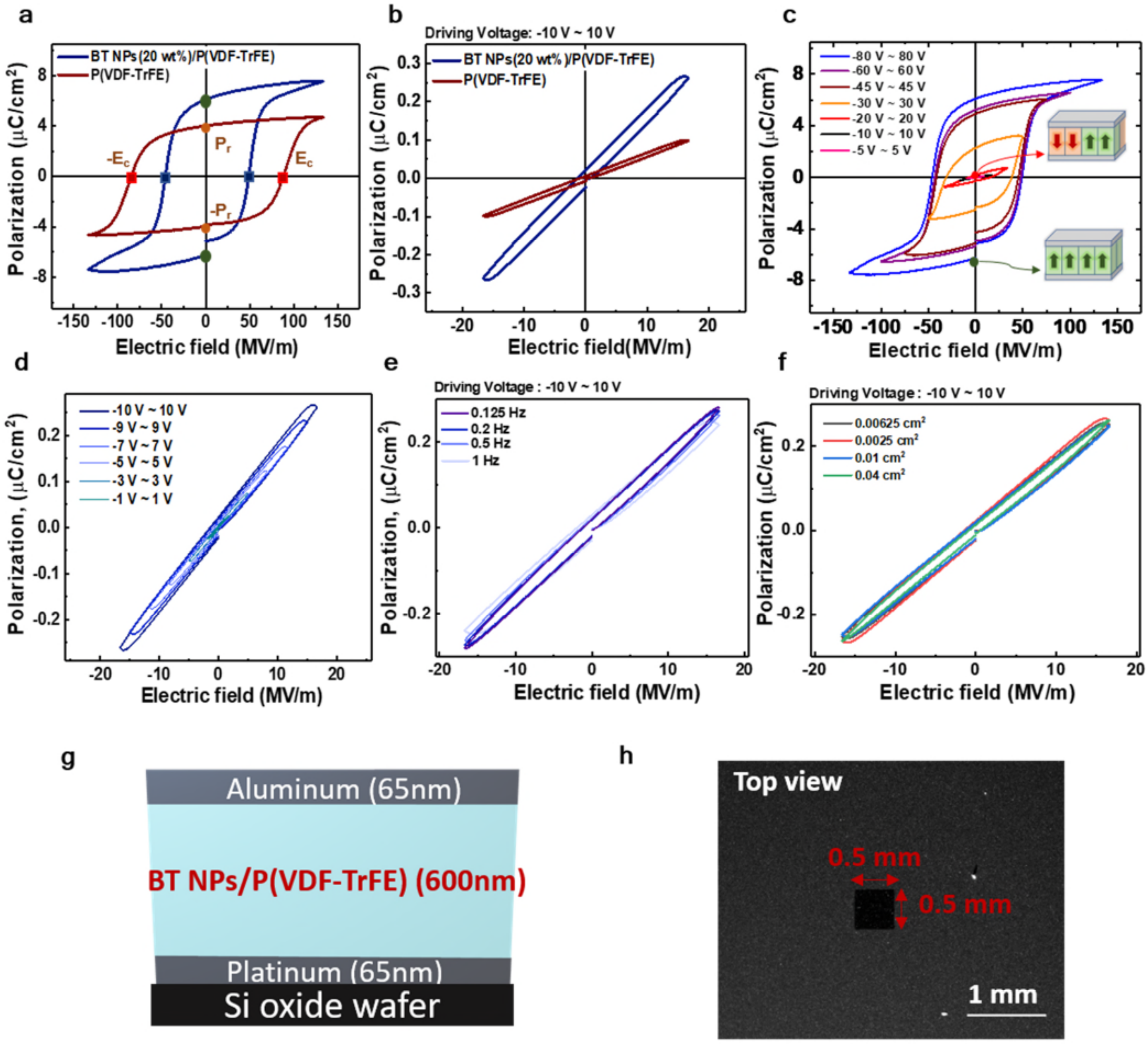


**Supplementary Figure 7. Fundamental characteristics of polarization-electric field (P-E) curve of ferroelectric films. a**, P-E curves of BT NP(20 wt%)/P(VDF-TrFE) and P(VDF-TrFE) thin films with the thickness of 600 nm. **b**, P-E curves of BT NP(20 wt%)/P(VDF-TrFE) and P(VDF-TrFE) thin films by applying -10 V to 10 V as driving voltage. **c**, P-E curves of BT NP(20 wt%)/P(VDF-TrFE) nanocomposite thin film with the applied voltage varied. The sub-loops of P-E at the applied voltage range of -5 V ~ 5 V to -60 ~ 60 V were also included. **d**, The sub-loops of P-E curve of BT NP(20 wt%)/P(VDF-TrFE) nanocomposite thin film at the applied voltage range of -1 V ~ 1 V to -10 V ~ 10 V. P-E curve of BT NP(20 wt%)/P(VDF-TrFE) nanocomposite thin film at the applied voltage range of -10 V to 10 V with the **e**, frequency varied of applied voltage **f**, and electrode area of devices. **g**, Schematic of device structure for measurement of P-E curve as MIM (metal-insulator-metal) structure. **h**, Optical image of MIM device at top

view.

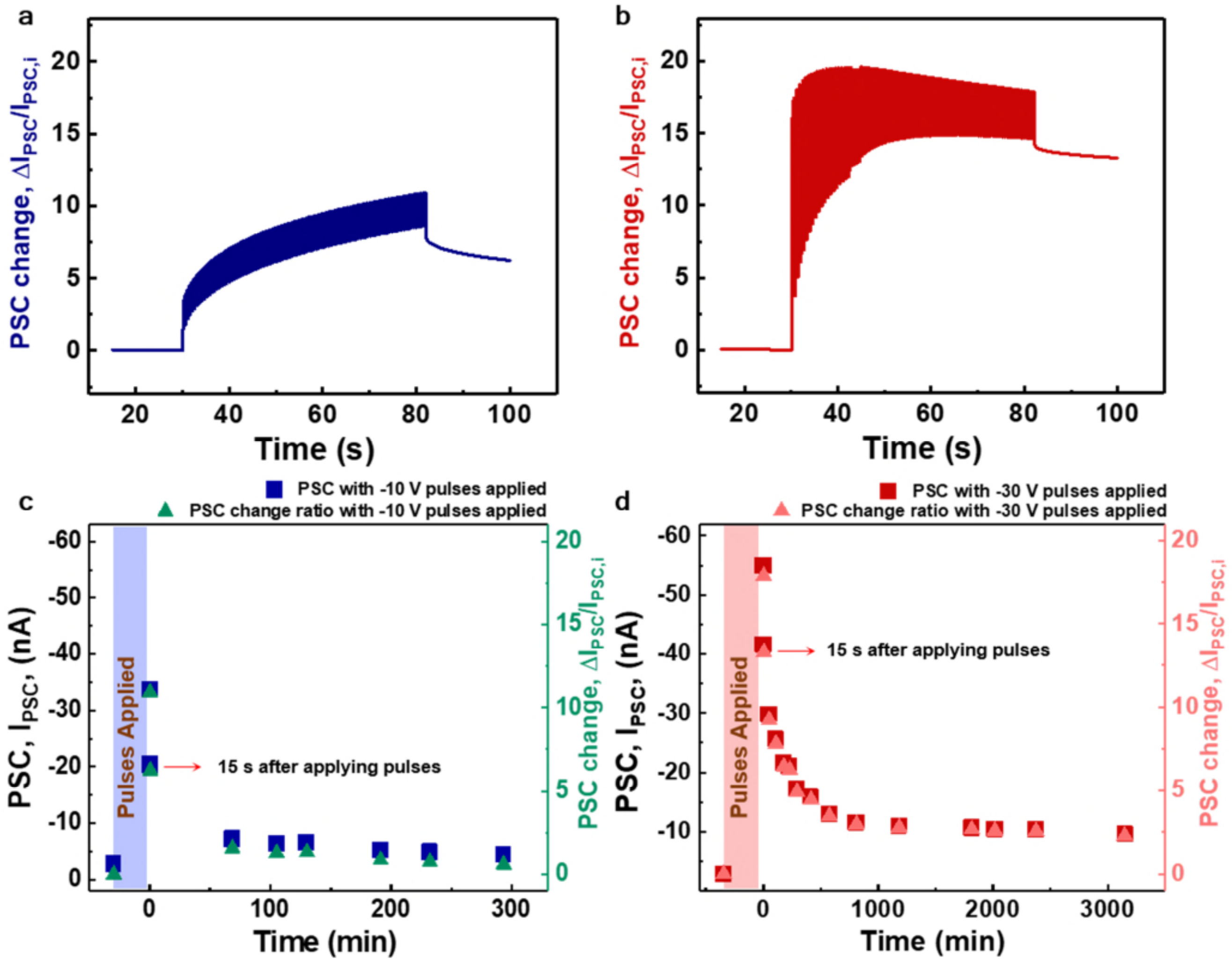


**Supplementary Figure 8. Comparison PSC and retention time with applying pulse of -10 V and -30 V of $V_{rec}$. a,** PSC change when applying the 100 pulses of $V_{rec}$ (amplitude of -10 V, pulse width of 0.5 s). **b,** PSC change when applying the 100 pulses of $V_{rec}$ (amplitude of -30 V, pulse width of 0.5 s). The retention time of PSC and PSC change after pulsing of **c**, -10 V and **d**, -30 V of $V_{rec}$.

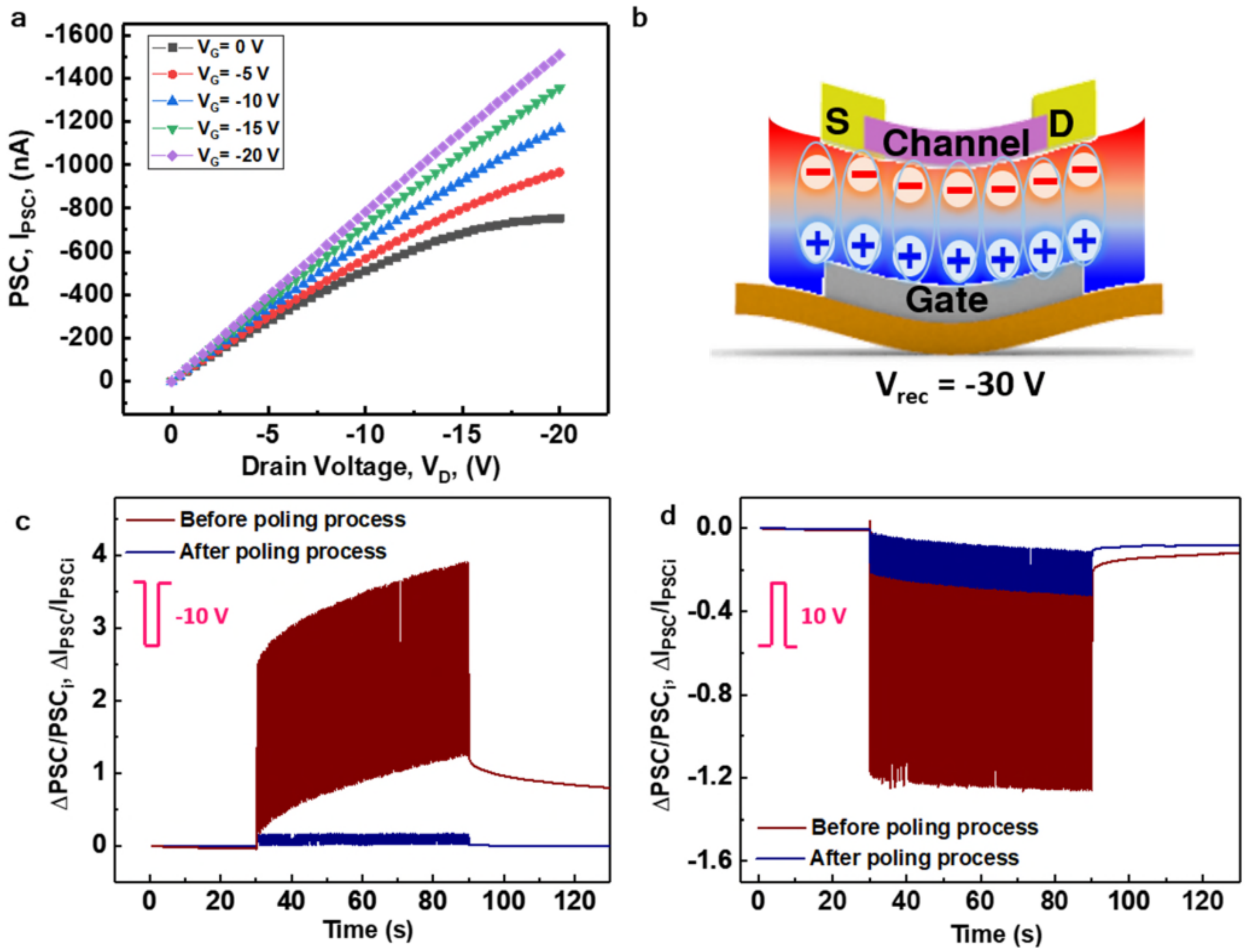


**Supplementary Figure 9.** Characteristics of Fe-OFET after poling process and comparison of PSC values before and after device poling. **a**, Output characteristics of poled Fe-OFET (using BT NPs 20 wt%/P(VDF-TrFE) composite as gate insulator). The poling was carried out by applying the negative gate bias of -30 V for 30 min. **b**, Dipole switching after poling process by applying the field between gate and drain electrode and formation of internal field. PSC change ratios of unpoled (red) and poled (blue) Fe-OFET by applying 100 pulses of $V_{rec}$ with the amplitude of **c**, -10 V and **d**, 10 V (pulse width of 0.5 s).

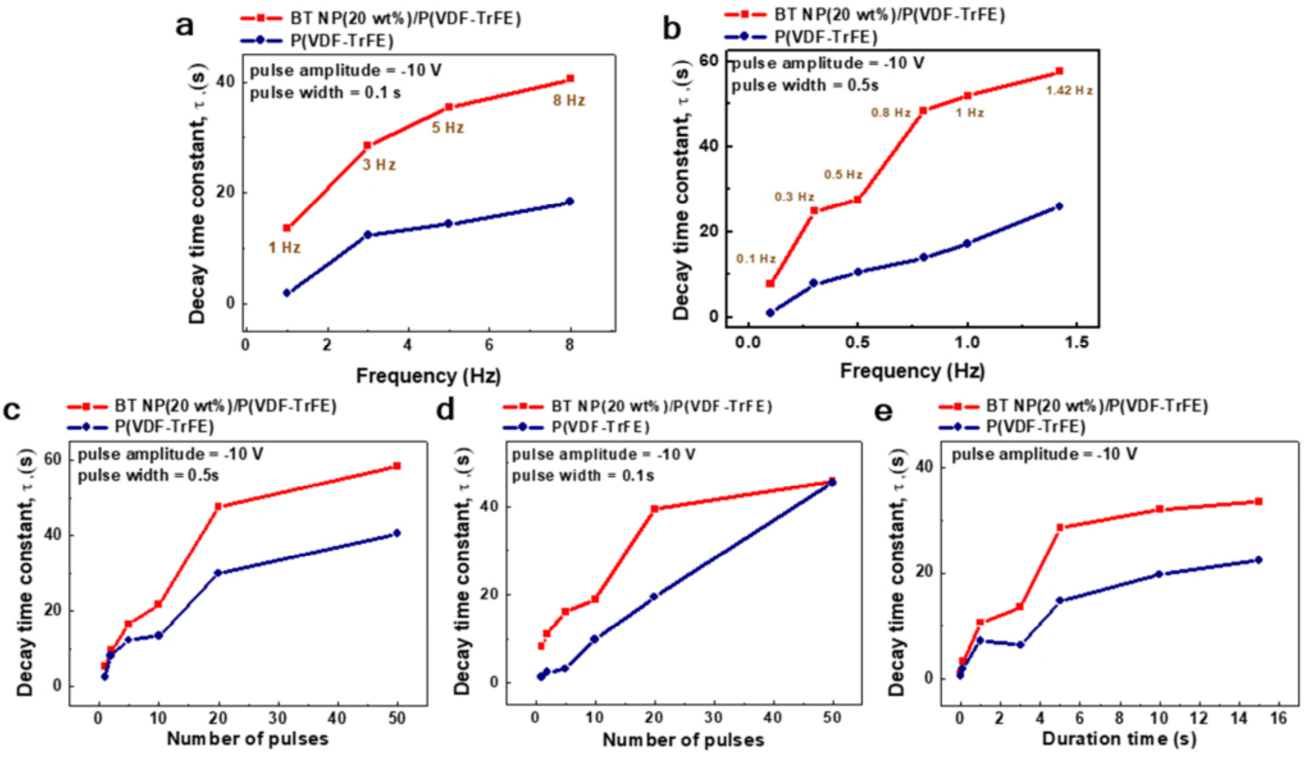


**Supplementary Figure 10. Analysis of decay time constants.** Decay time constants, τ, of devices with BT NP(20 wt %)/P(VDF-TrFE) (red) or P(VDF-TrFE) (blue) **a,** at different frequencies of $V_{rec}$ (pulse width of 0.5 s and amplitude of -10 V), **b**, different frequencies of $V_{rec}$ (pulse width of 0.1 s and amplitude of -10 V), **c**, different number of pulses of $V_{rec}$ (pulse width of 0.5 s and amplitude of -10 V), **d**, different frequencies of $V_{rec}$ (pulse width of 0.1 s and amplitude of -10 V) and **e**, different pulse durations of $V_{rec}$ (amplitude of -10 V).

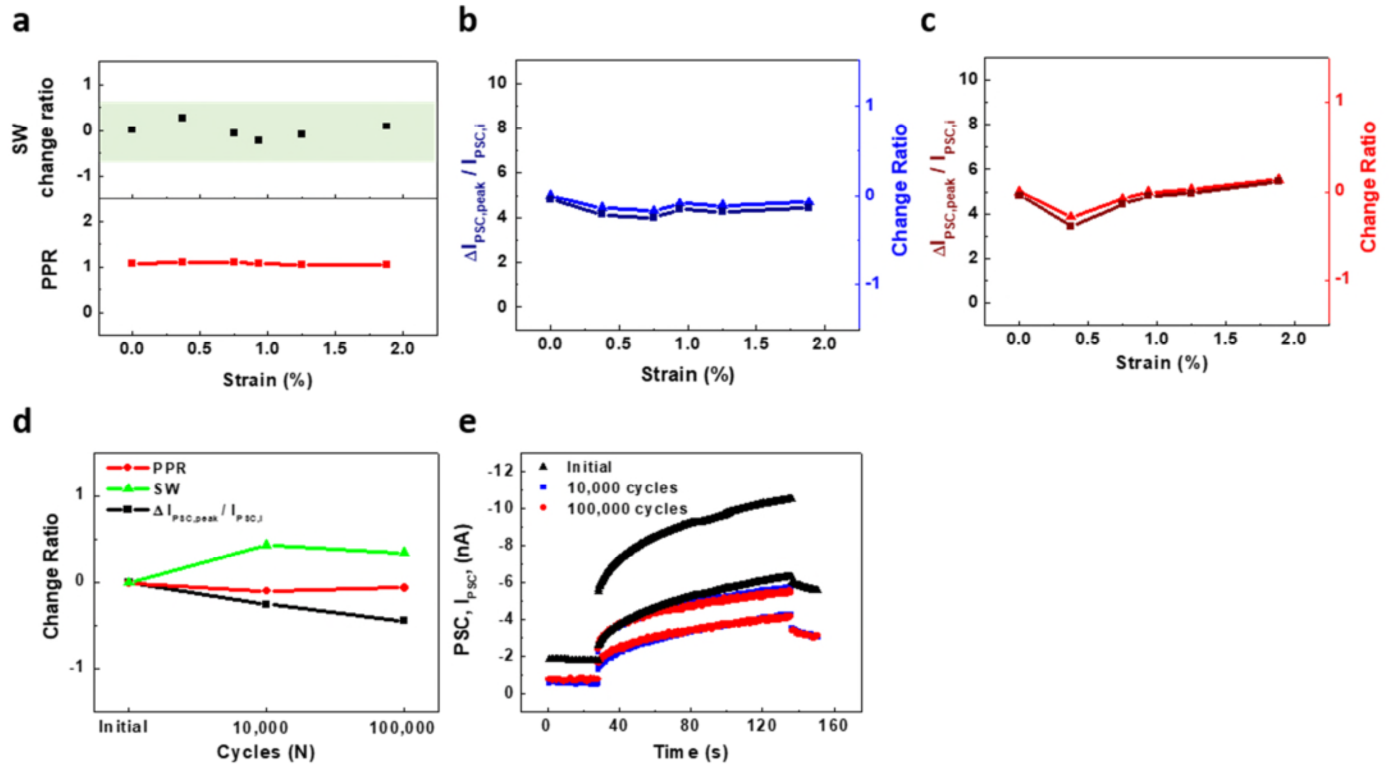


**Supplementary Figure 11. Mechanical flexibility test of Fe-OFETs under tensile and compressive strain. a**, Variations in SW change ratio and PPR under different static compressive strains (two successive pulses with a pulse width of 0.5 s and amplitude of -10 V). **b, c,** Increased amount and variation of peak PSC values (pulse width of 0.5 s and amplitude of -10 V) for different **b**, tensile and **c**, compressive static strains (0.375%, 0.75%, 0.9375%, 1.25%, and 1.88%). **d**, change ratio of PPR and increase in peak PSC and SW under a dynamic tensile strain of 1.25%. **e**, PSC with repetitive pulses around 100 s (pulse width of 0.1 s and amplitude of -10 V) before and after 10,000 and 100,000 cycles of exposure to a tensile strain of 1.25%. The cyclic bending test results indicate that there is a significant difference in the $I_{PSC}$ values between initial state and after compressive bending of 10,000 cycles (**Supplementary Figure 9e**). The $I_{PSC}$ values after 10,000 or 100,000 cycles are not much different even the number of bending cycles increases more than that between the initial and 10,000, which indicate that there is a stabilization stage similarly to other organic flexible devices.[1–5] AiS-TSO are not mechanically stretchable but flexible. There are some limitations in mimicking deformability of the skin for applications in electronic skin or soft robotics but its flexibility has many advantages compared to rigid devices.

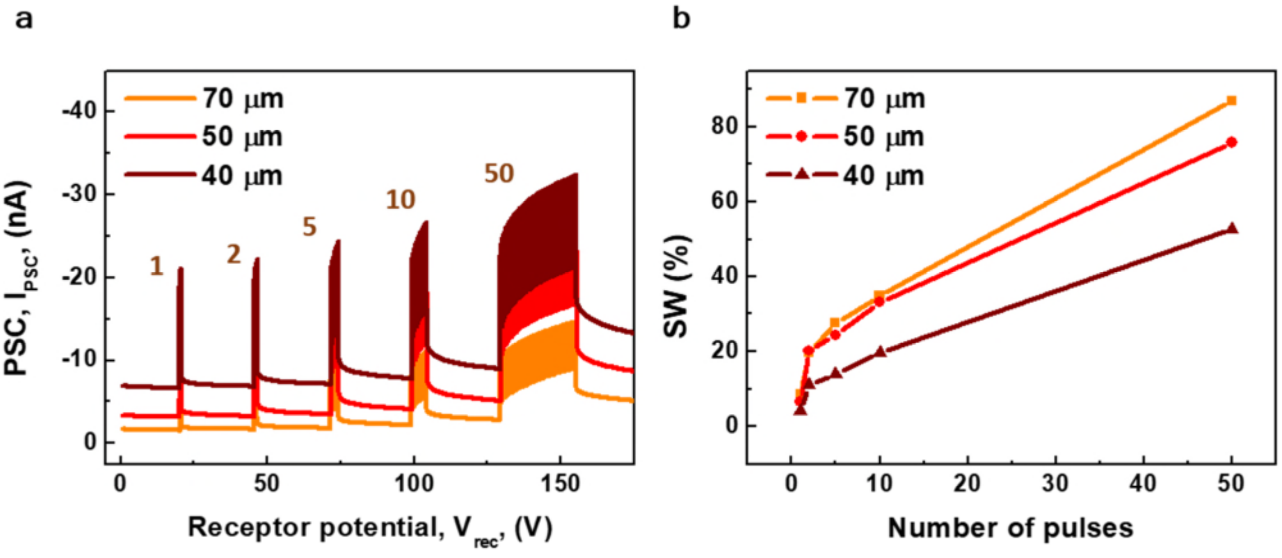


**Supplementary Figure 12. Characteristics of Fe-OFET depending on channel length. a**, $I_{PSC}$ with varying the number of $V_{rec}$ pulses from 1 to 50 from Fe-OFETs with channel length of 40, 50 and 70 μm (pulse width of 0.2 s and amplitude of -10 V). **b**, synaptic eight (SW) analysis depending on the channel length and number of $V_{rec}$ pulses. To investigate the channel length effect of Fe-OFET, we fabricated the device with different channel lengths of 40, 50 and 70 μm. As shown in **Supplementary Figure 10a**, the $I_{PSC}$ level was increased with the channel length decreased, as expected. On the other hand, as shown in **Supplementary Figure 10b**, SW increases as the channel length increases. This observation can be explained as an effect of increased retention time with the channel length increased which is related with the gate area of Fe-OFET[6–11] and slower switching time of dipoles in ferroelectric gate dielectric of Fe-OFET[8,10,12].

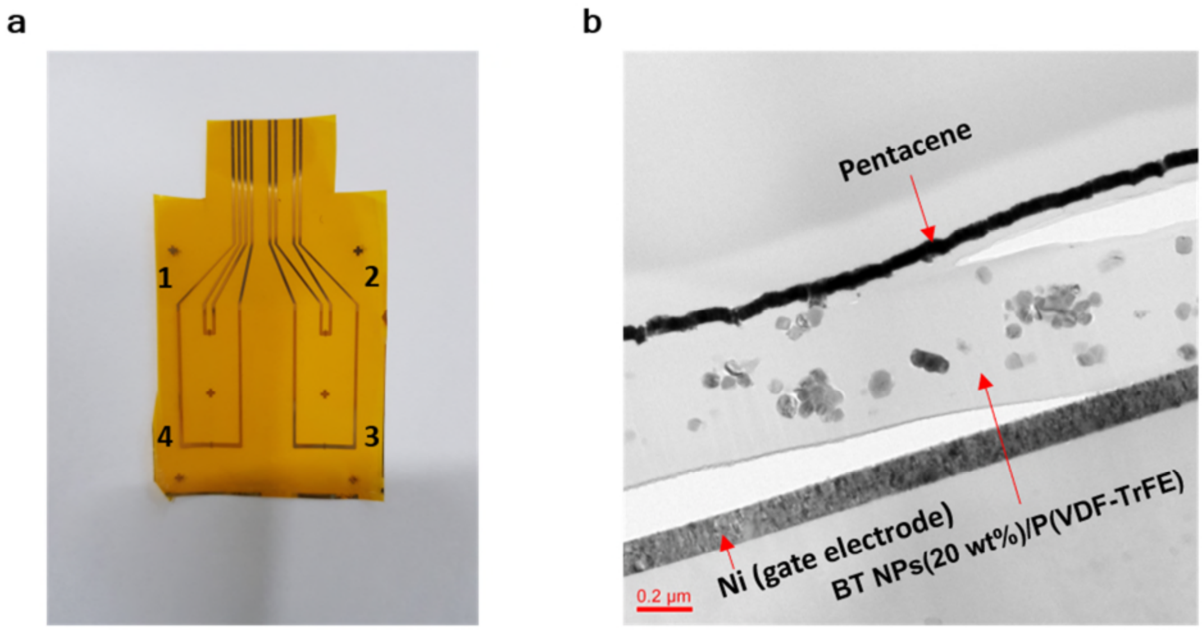


**Supplementary Figure 13. Image of AiS-TSO and channel area of Fe-OFET. a**, photograph of AiS-TSO with numbering the pixel. **b**, cross-sectional TEM (transmission electron microscopy) image of Fe-OFET in channel area prepared from the device structure by FIB (focused ion beam). The gap between Ni electrode and gate insulating layers was originated from delamination during sample cutting by FIB.

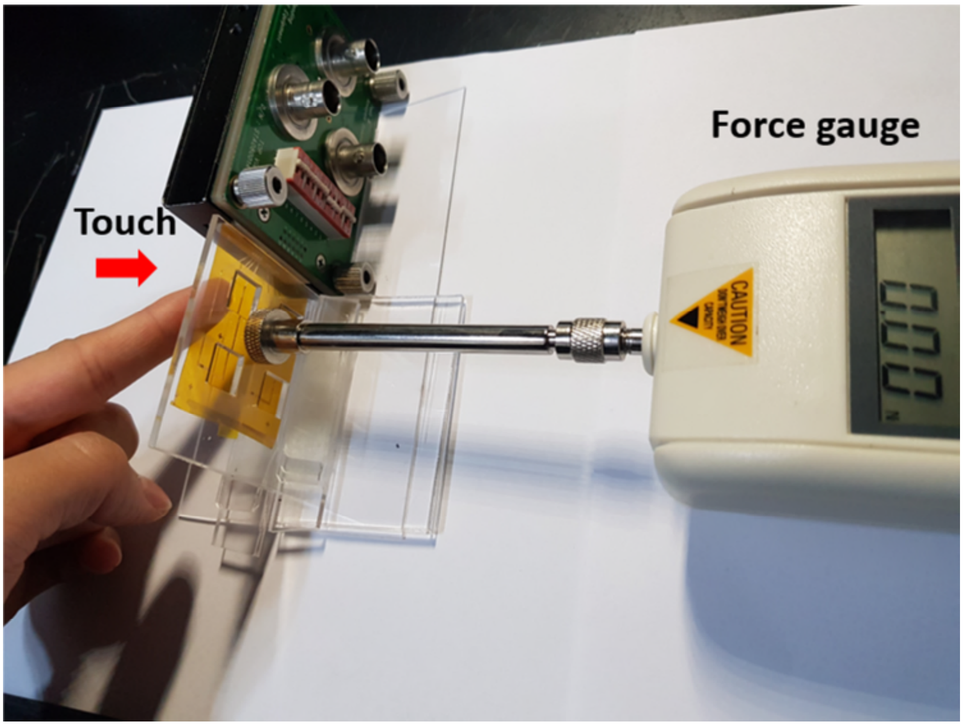


**Supplementary Figure 14. Measurement setting for responses of AiS-TSO to touch.** Measurement environment with force gauge and PCB board connected with the device.

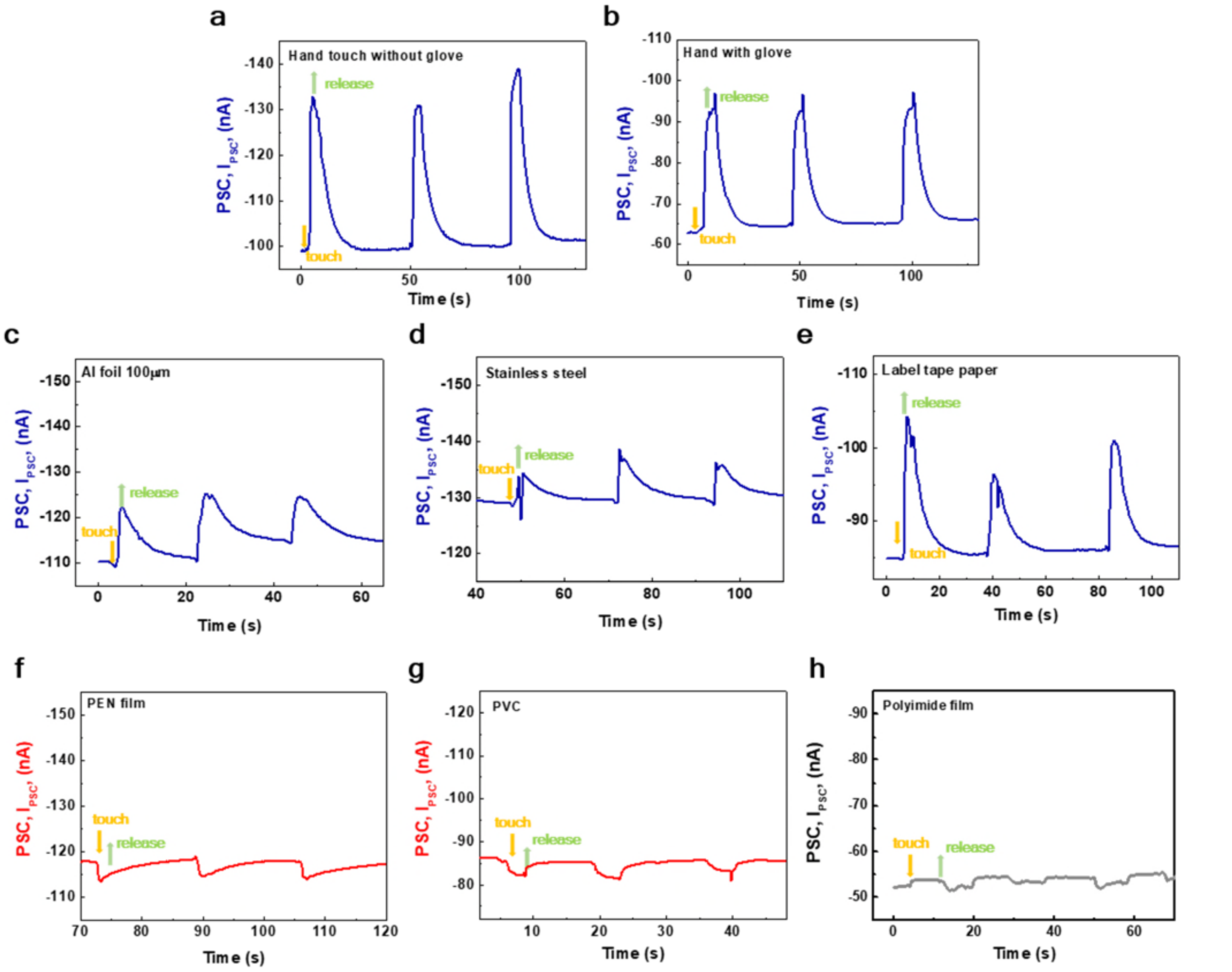


**Supplementary Figure 15. PSC dependence on touched materials.** PSC when the **AiS**-TSO was touched with different materials (**a**, bare hand, **b**, gloved hand, **c**, aluminum foil, **d**, stainless steel foil, **e**, label tape paper, **f**, polyethylene naphthalate (PEN) film, **g**, polyvinyl chloride (PVC) film, **h**, polyimide film with a force of ≈ 1 kPa.

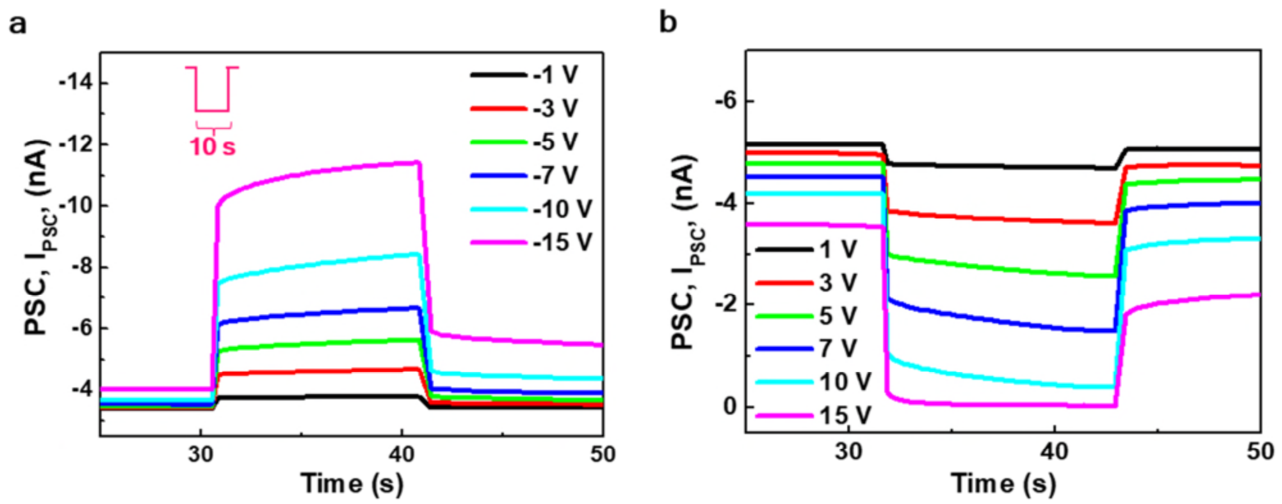


**Supplementary Figure 16. Slowly adapting reception property of the Fe-OFET.** $I_{PSC}$ was recorded for $V_{rec}$ pulses (duration of 10 s) applied with **a,** a pulse amplitude of -10 V for potentiation and **b**, a pulse amplitude of 10 V for depression.

$I_{PSC}$ increased (decreased) when a negative (positive) $V_{rec}$ was applied, but the ratio of the increase (decrease) decreased, which is a slow adaptation (SA) property that results from continuous changes in ferroelectric polarization that is similar to the SA characteristics of Merkel-cell membrane potential.

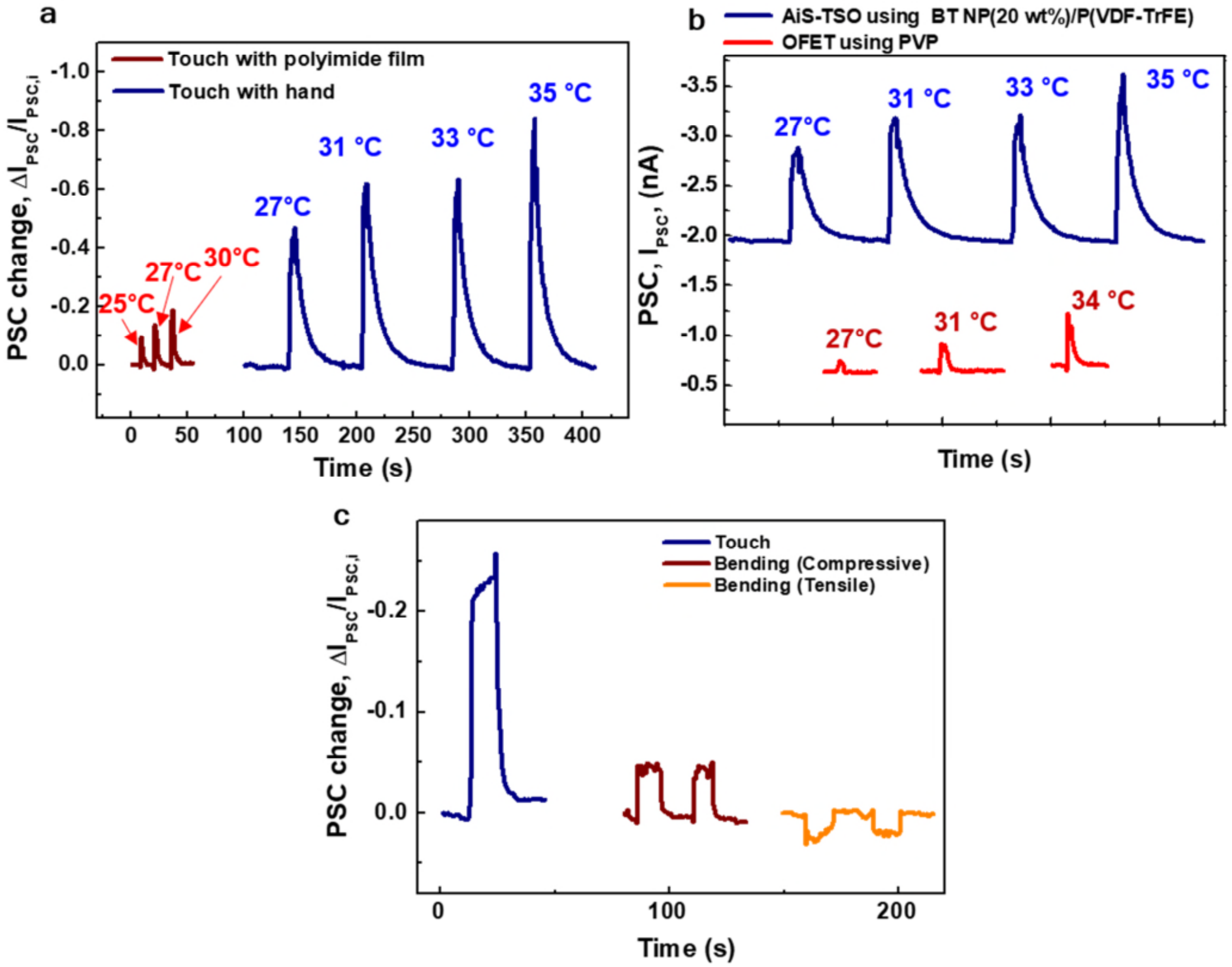


**Supplementary Figure 17. Responses of AiS-TSO to thermal and strain stimuli. a**, change ratio of PSC ($\Delta I_{PSC}/ I_{PSC,i}$) of AiS-TSO when touched by polyimide (PI) film and finger with varying temperature of polyimide and finger. The devices were touched with stimulation time of ≈3s at the force of≈ 1 kPa. **b**, PSC ($I_{PSC}$) of AiS-TSO with BT NP(20 wt%)/P(VDF-TrFE) gate dielectric (blue) and OFET with PVP gate dielectric (red) with the temperature of finger varied during finger touch. The devices were touched by finger with stimulation time of ≈3s at the force of≈ 1 kPa. **c**, change ratio of PSC ($\Delta I_{PSC}/ I_{PSC,i}$)in AiS-TSO under touching (stimulation time of ≈10 s at the force of ≈1 kPa) and bending strains (bending radius of 2 mm).

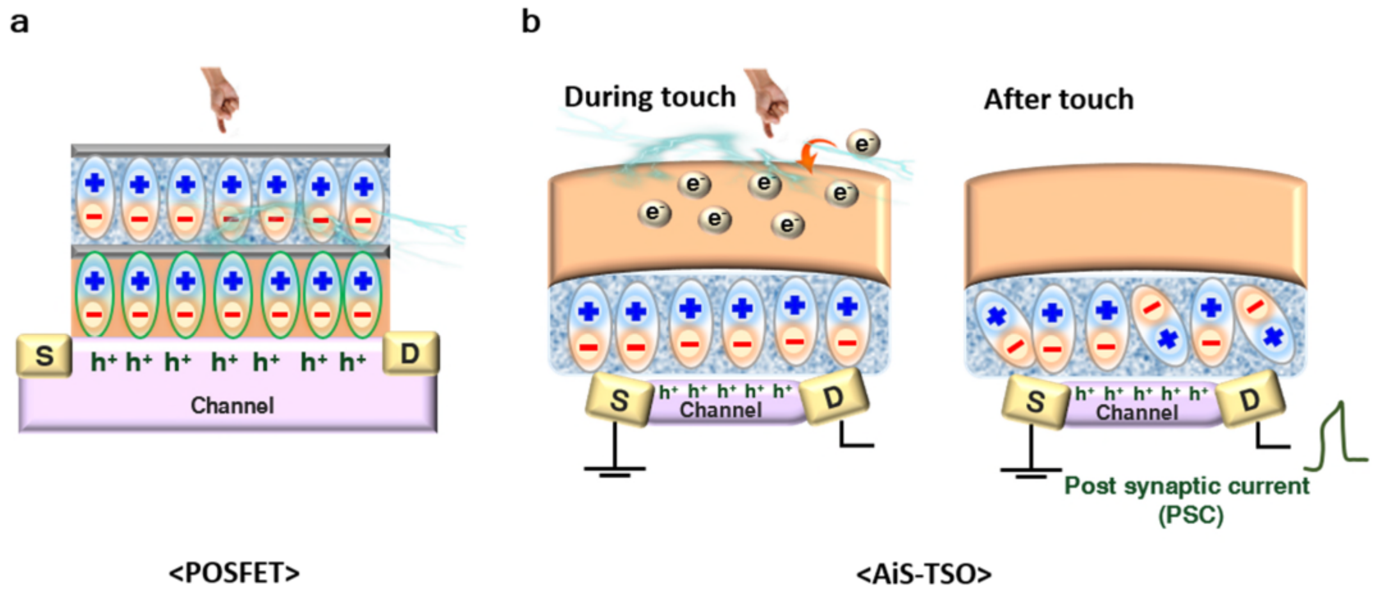


**Supplementary Figure 18. Comparison between piezoelectric oxide semiconductor FET (POSFET) and AiS-TSO as touch sensor.** Schematic illustration of working principle of **a**, POSFET tactile sensor and **b**, AiS-TSO during touch (left) and after touch (right).

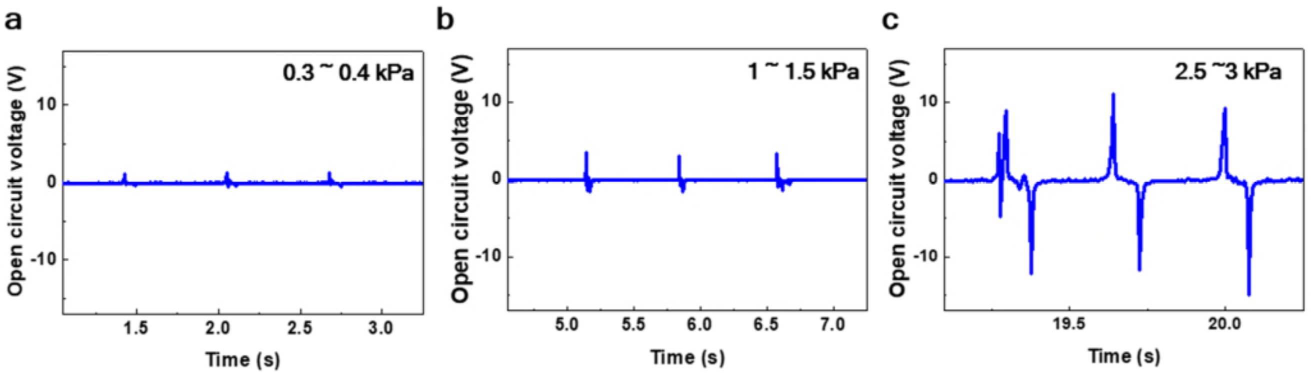


**Supplementary Figure 19. Open circuit voltage of AiS-TSO according to touching force.** Open circuit voltage output from triboelectrification induced by contact between a finger and polyimide film with different force ranges of **a**, 0.3~0.4 kPa, **b**, 1~1.5 kPa, and **c**, 2.5~3 kPa. Force applied by finger touch was adjusted using a hand force gauge.

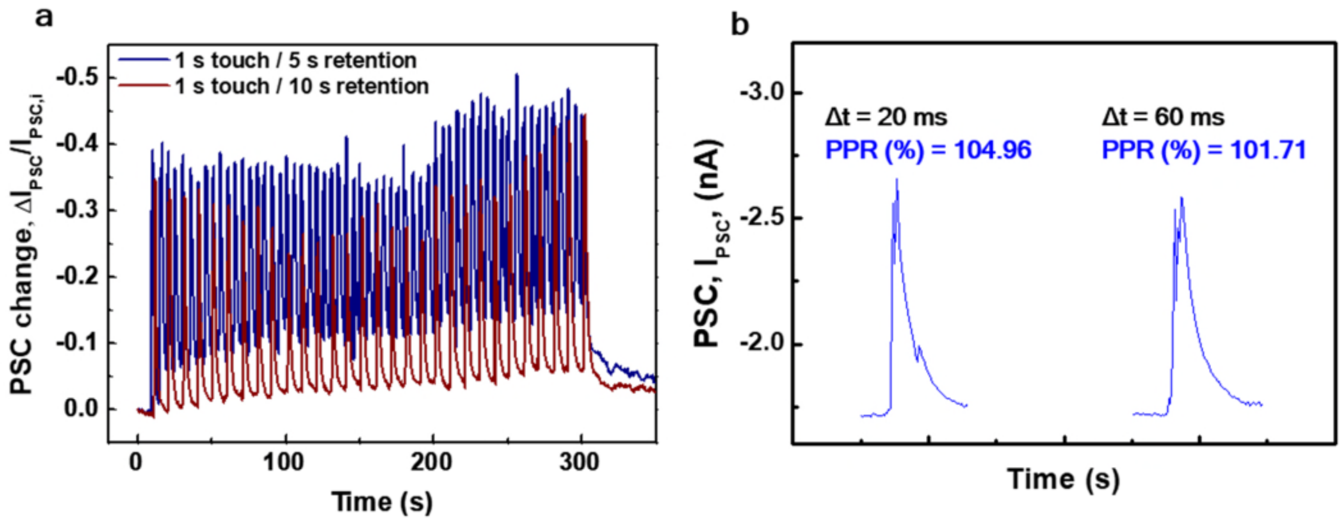


**Supplementary Figure 20. Characteristics of AiS-TSO with repetitive touches and different number of touches**. **a**, Change ratio of PSC ($\Delta I_{PSC}/I_{PSC,i}$)with repetitive touches having retention time of 1 s and retention time of 5s and 10 s. **b**, $I_{PSC}$ with consecutive touches depending on different time interval and PPR value. All the data were obtained at the touch force of ≈ 1 kPa.

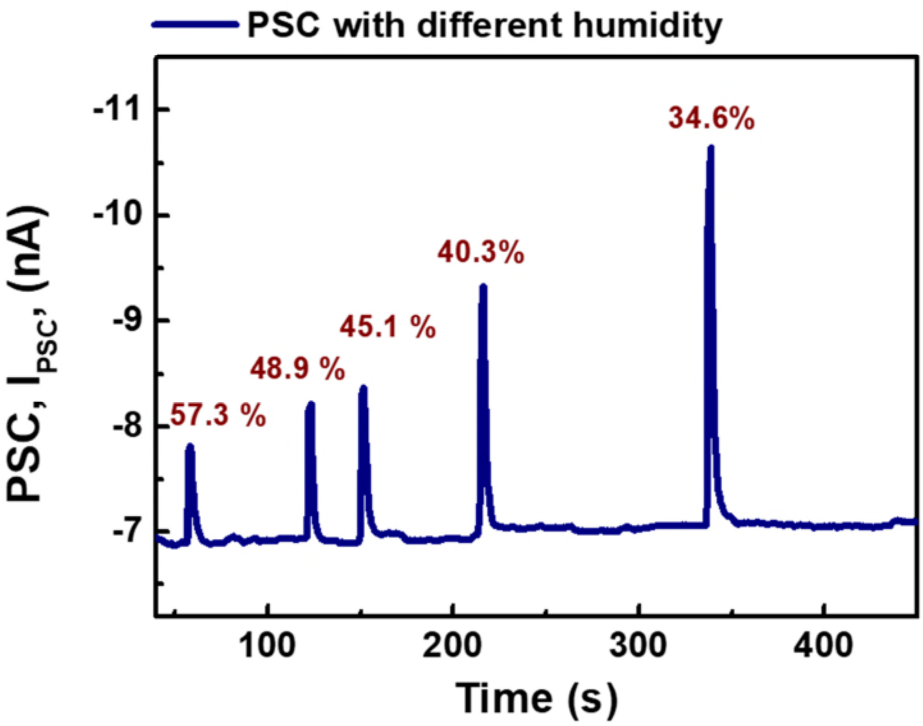


**Supplementary Figure 21. Measurement of AiS-TSO varying humidity condition.**

Here we touched the AiS-TSO varying the humidity condition with around 1 kPa pressure. As shown in the **Supplementary Figure 19**, the response was decreasing with humidity increasing. This phenomenon can be explained the humidity effect on triboelectricity. Since the polyimide film is hydrophilic, so water absorption is high when it is in the high humidity condition increasing surface conductivity from water layer[13]. The higher surface conductivity discharges the surface decreasing the effective triboelectric charges transfer between polyimide film and skin[14].

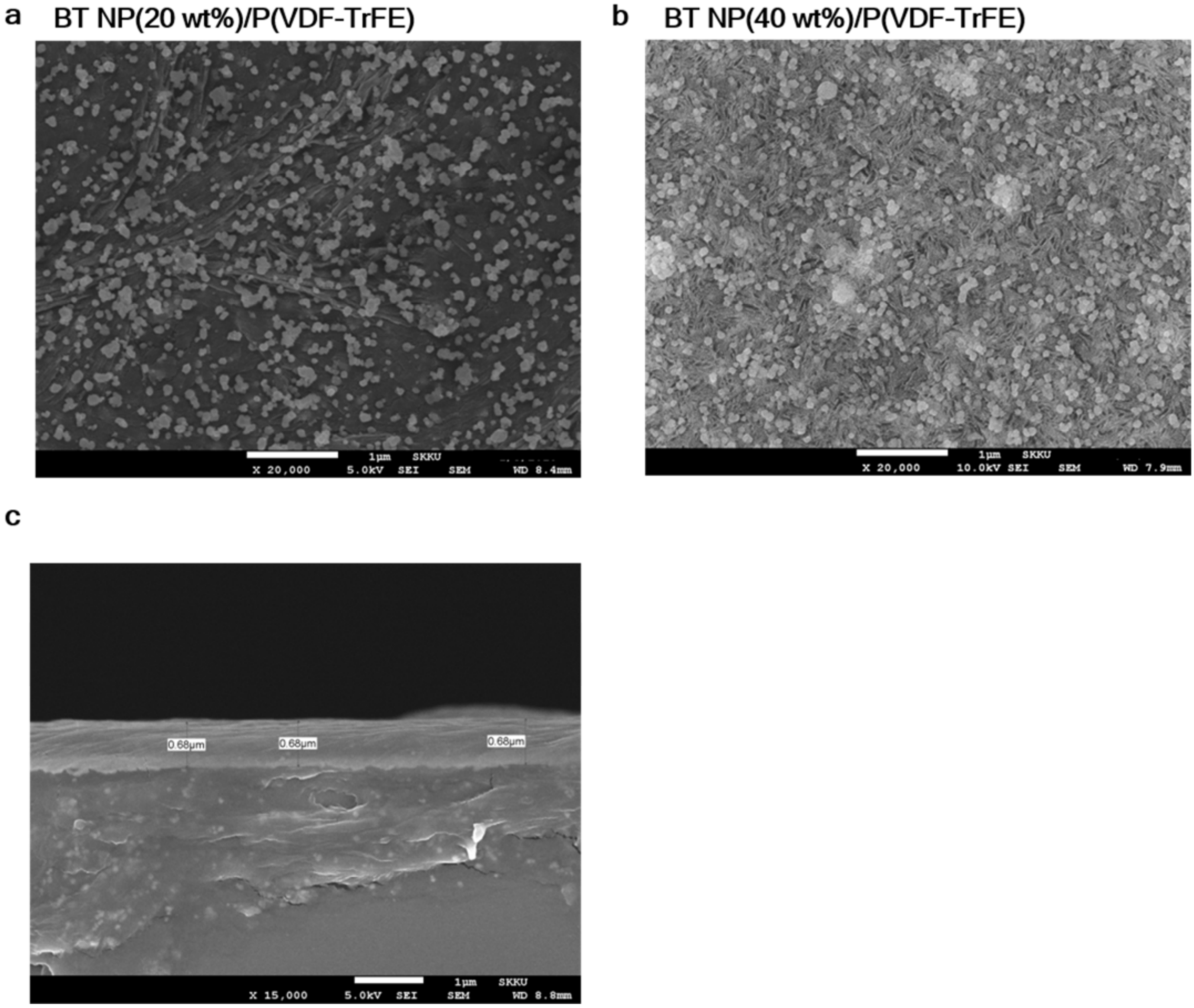


**Supplementary Figure 22. FE-SEM images of ferroelectric nanocomposites.** FE-SEM images of ferroelectric nanocomposites of **a**, BT NP(20 wt%)/P(VDF-TrFE), **b**, BT NP(40 wt%)/P(VDF-TrFE) and **c**, cross-sectional FE-SEM image of the nanocomposite of BT NP(20 wt%)/P(VDF-TrFE) coated on Si wafer to confirm thickness of thin film layer. The thickness was estimated around 0.6 μm. The scale bar in all images is 1 μm.

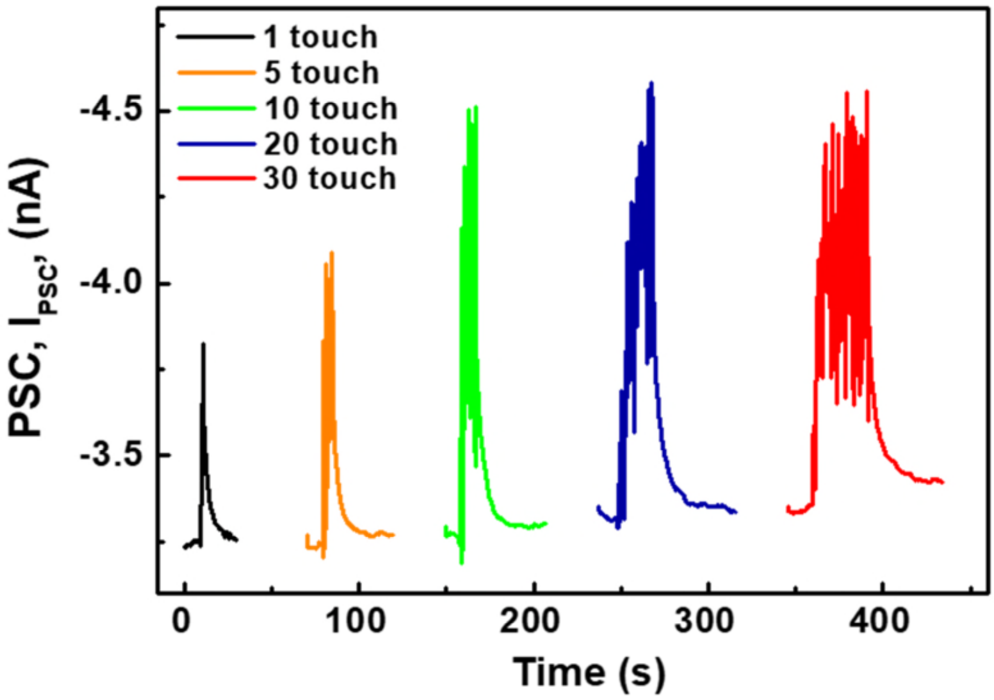


**Supplementary Figure 23. $I_{PSC}$ with the number of touches varied.** Touch force of ≈ 1 kPa varying number of touches.

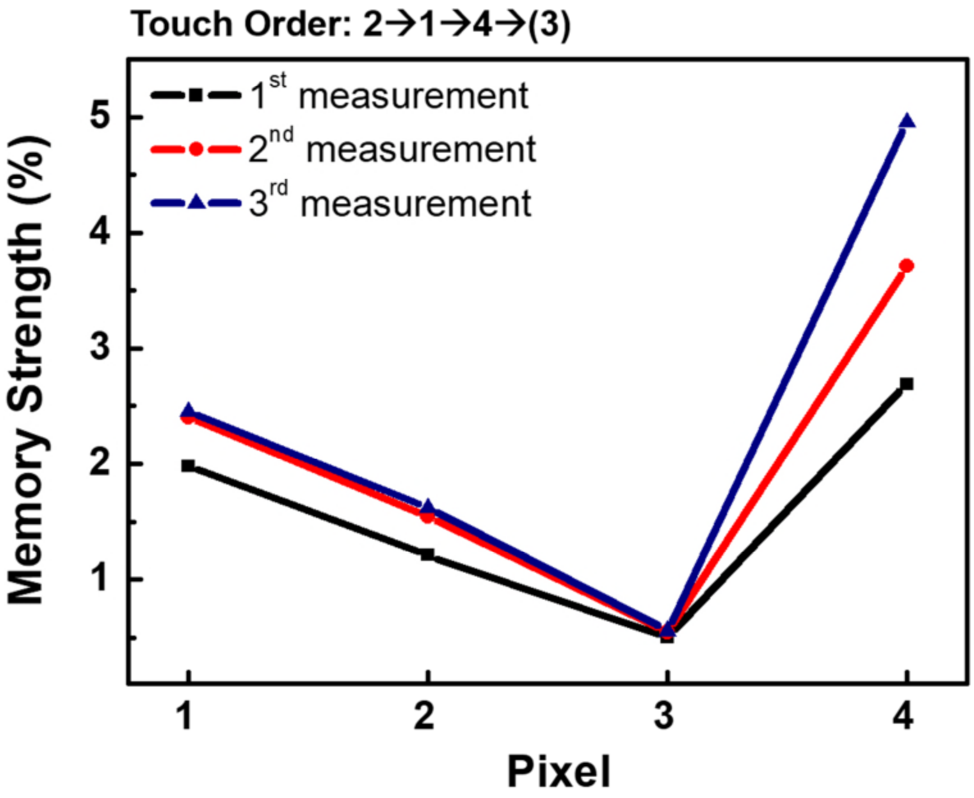


**Supplementary Figure 24. Measurement of memory strength to demonstrate memory-embedded function in AiS-TSO upon finger touches.**

Here, we measured the memory strength of AiS-TSO in an order of touch, pixel 2→1→4→(3) while the pixel 3 was skipped (not touched). The measurements were repeated three times. All three experiments showed the same tendency that the untouched pixel 3 has the smallest memory strength and the memory strength was increased from the firstly touched (pixel 2) to lastly touched device (pixel 4).

**Supplementary Table**

| Merkel Cell - Neurite Complex (MCNC) | Artificial intrinsically Intelligent – Tactile Sensory Organ (AiS-TSO) |
| --- | --- |
| Piezo-2 channel gating | Mechanical triboelectric gating |
| Merkel cell membrane | Triboelectric layer |
| Neurotransmitter | BT NP/P(VDF-TrFE) dipoles polarization |
| Post synaptic current | Drain current |
| Synaptic weight | Inherent conductivity change |

**Supplementary Table 1. Comparison of a Merkel-cell neurite complex (MCNC) and artificial intrinsic-intelligence tactile sensory organ (AiS-TSO).**

## Supplementary Note

### Supplementary Note 1

The analogy between an MCNC and AiS-TSO is explained for each step of signal propagation described in **Fig. 1**.

Step (i): Tactile stimulation is applied.

Step (ii): In an MCNC, Merkel cells, which are mechanical sensory epithelial cells that have mechanically activated Piezo-2 ion channels, activate a SA-I neuronal afferent. Upon tactile stimulation, $Ca^{2+}$ enters the cell via Piezo-2 ion channels and increases membrane potential[15,16]. In our AiS-TSO, tactile stimulation generates a triboelectric potential due to influx of electrons from triboelectrification between the finger and receptive polyimide layer, which corresponds to an artificial Merkel cell membrane. Similar to the MCNC, the AiS-TSO mechanism uses mechanical energy to generate a potential without any external energy.

Step (iii): Merkel cells have a high membrane resistance; therefore, the $Ca^{2+}$ ion current generated by influx of $Ca^{2+}$ through the Piezo-2 channels produces a large sustained depolarization of the Merkel cell[17]. The ability to produce sustained depolarization via Piezo-2 channels is responsible for the two-receptor-site mechanism where inactivation of the Piezo-2 channel results in SA firing[17,18]. By analogy, the receptive polyimide layer of our AiS-TSO, which corresponds to an artificial Merkel cell membrane, has an ability to store electrons from triboelectrification and therefore functions as a capacitor to generate a SA output signal. The rate of increase in potential of the polyimide layer decreases gradually, which means that the response to the stimulus gradually decreases, and SA reception is achieved.

Step (iv): In an MCNC, Merkel cells act as pre-synaptic neurons and release serotonic neurotransmitters to activate receptors of A□ afferents for excitatory signals[19]. Similarly, the potential generated in the receptive polyimide layer of the AiS-TSO switches the dipoles of the BT NP/P(VDF-TrFE) nanocomposite ferroelectric layer above the coercive field. Polarization changes due to dipole switching in the AiS-TSO correspond to neurotransmitter release in the MCNC.

Step (v): In an MCNC, the amount of neurotransmitter that accumulates depends on the degree of tactile stimulation, resulting in synaptic plasticity, which, in turn causes generation of an action potential in the A□ afferent to transduce SA-I signaling[18,19]. In our AiS-TSO, the amount of permanent polarization depends on the degree of stimulation as this modulates the drain current, i.e., post synaptic current ($I_{PSC}$) and can be considered the synaptic weight. Indeed, the retention time of synaptic weight in a biological MCNC is much shorter than that in the AiS-TSO although the exact principles of sensory memory function in an MCNC have not yet been elucidated. In our AiS-TSO, short-term and long-term plasticity (STP and LTP) can form because of the ferroelectric properties of the gate dielectric layer, which makes it possible to create short-term and long-term memory in addition to sensory memory. In an MCNC, A□ afferents convert the postsynaptic current to action potentials depending on the strength of the synaptic connection. Our AiS-TSO mimics the synaptic connections and functions of an MCNC prior to converting the PSC to an action potential; and PSC is endowed with synaptic weight which can be tuned by varying the composition of the nanocomposite ferroelectric gate dielectric layer.

**Supplementary Note 2**

When the frequency of the receptor potential was low, the change ratio of $I_{PSC}$ for a pulse width of 0.1 and 0.5 s exhibited STP properties, while for faster pulses, the $I_{PSC}$ exhibited LTP properties, both of which are consistent with biological synapse properties. For a pulse frequency of 1 Hz and pulse width of 0.5 s, $I_{PSC}$ showed LTP tendencies **(Supplementary Figure 2a),** but at a pulse width of 0.1 s, STP properties were observed **(Supplementary Figure 2b)**. Pulse width (duration) is also an important determinant of STP or LTP. Our data indicate that STP or LTP characteristics can be obtained by regulating frequency and duration, which are related to the SRDP and SDDP of biological synapses, respectively.

**Supplementary Note 3**

To demonstrate that ferroelectric characteristics of gate dielectric layer in Fe-OFET mainly contribute to synaptic properties of the device, we also fabricated the OFET with non-ferroelectric PVP (polyvinylpyrrolidone) as a gate dielectric layer. The measured capacitance of BT NP(20wt%)/P(VDF-TrFE) was much higher (~21 nF/cm$^2$ , dielectric constant = 13.81) than PVP (~5.2 nF/cm$^2$, dielectric constant = 3.25) in the MIM structure with the same insulator thicknesses as those in the FET structure, which results in the higher on-state current level in the transfer characteristics (**Supplementary Figure 3a**) in Fe-OFET with ferroelectric nanocomposite than that in OFET device with PVP device. The change of PSC (ΔPSC) of the device with PVP (~1x10$^{-11}$ A) during $V_{rec}$ biasing was much smaller than that of the device with BT NP(20 wt%)/P(VDF-TrFE) (~1x10$^{-9}$ A) during $V_{rec}$ biasing (**Supplementary Figure 3b**). However, Fe-OFET with ferroelectric gate dielectric material has larger hysteresis in the transfer curve (**Supplementary Figure 3a**) and larger change in PSC after finishing $V_{rec}$ pulsing (**Supplementary Figure 3b**) compared to OFET with PVP gate insulator. These results are originated from internal field generated in partial polarization switching in ferroelectric material, which results in the generation of synaptic weight (SW) in the Fe-OFET. On the other hand, negligible hysteresis and residual ΔPSC after finishing $V_{rec}$ pulsing in the OFET with non-ferroelectric PVP indicate that charge trapping does not significantly affect synaptic properties as much as ferroelectric effect event though charge trapping of organic semiconductors has been reported.[20,21] On the other hand, it can be argued that synaptic property in our Fe-OFET device is mainly related to ferroelectricity of nanocomposite gate dielectric layer.

**Supplementary Note 4**

To demonstrate the repeatability of Fe-OFET as a synaptic device, we applied the $V_{rec}$ of increasing and decreasing amplitude of pulses and number of pulses. As shown in **Supplementary Figure 6**, PSC response was almost the same when we applied the $V_{rec}$ in increasing or decreasing amplitude and pulse number. Since we applied each pulse after full recovery to the state at the previous pulse, PSCs were only minimally affected by the previously formed polarization. Therefore, we found that if we need the repeatability of the

device, we can apply the pulse after the full recovery. Here the repeatability in SW was good when we increase and decrease the amplitude of pulses and the number of pulses.

**Supplementary Note 5**

To confirm the effect of BT NPs in the nanocomposite film on polarization behaviors, we fabricated the metal (Pt)-ferroelectric-metal (Al) (MFM) structures with the ferroelectric layers of BT NP(20 wt%)/P(VDF-TrFE) nanocomposite and P(VDF-TrFE) on $SiO_2$/Si wafer and measured polarization-electric field (P-E) curves by applying the voltage from -80 V to 80 V (**Supplementary Figure 7a**). The smaller coercive field ($E_c$) of 48.83 ($-E_c$ = -45.83) MV/m for BT NP(20 wt%)/P(VDF-TrFE) nanocomposite film was obtained compared to that ($E_c$ = 88.2 MV/m, $-E_c$ = -84.6 MV/m) for pure P(VDF-TrFE) film. Also, the nanocomposite film has much larger remnant polarization ($P_r$) (6.1 μC/cm$^2$) than that of pure P(VDF-TrFE) (4 μC/cm$^2$). These results demonstrate that generation of larger $P_r$ in BT NP(20 wt%)/P(VDF-TrFE) was observed compared to pure P(VDF-TrFE), which means that synaptic behaviors of the Fe-OFET can be tuned by varying the concentration of BT NPs in the nanocomposite. There would be some differences in $P_r$ compared to the values from the previous reports about P(VDF-TrFE) thin films[22–25]. This could occur from different thickness of film, electrode area of device or measurement condition such as driving voltage.[8,10,11] However, the coercive field value was not much different from those in the previous reports and the tendency of increasing in polarization was observed when the BT NPs are included in nanocomposite, similarly to the results reported from the previous reports[22–26]. Also, when the range of applied voltage was varied to a smaller range, there was observed partial polarization in minor loop. We could demonstrate that usage of smaller range of voltage such as -10 V to generate SW by controlling the partial polarization with pulse rate, number and duration time varied. Therefore, polarization switching can be controlled according to the range of applied voltage[10,11,27–29], which implies that SW in Fe-OFET will depend on amplitude, duration time, rate and number of the $V_{rec}$ pulses similar to other Fe-RAM devices using minor loop of ferroelectric materials.[23,30–34] Of course, the device has smaller retention time for polarization of minor

loop[30–33]. However, the retention time can be controlled by pulse duration time, number or frequency for generating SW and controlling the STP and LTP behaviors, as shown in **Fig. 2**.

**Supplementary Note 6**

In **Supplementary Figure 8**, we checked the PSC values when applying -30 V of $V_{rec}$ which is above the coercive voltage of gate insulating layer in Fe-OFET. Comparing with the results obtained with -10 V of $V_{rec}$, the results show the fast saturation in PSC but poor characteristics in terms of linearity of PSC increasing rate. On the other hand, in case of retention time, the time taken to drop below 15% of the maximum PSC change at -30 V was longer (~1814 min) than that at -10 V (~68 min). Also, the maximum PSC increasing rate was also about 2 times at -30 V of $V_{rec}$ (~18) larger than that at -10 V of $V_{rec}$ (~11). This is related to remnant polarization formed by applying bias pulses above coercive field and, therefore, much longer retention time was obtained compared to that by applying bias pulses in minor loops of P-E curve. But as we mentioned before, we can generate and adjust SW even with -10 V of $V_{rec}$ pulses. Therefore, we judged that it was not necessary to apply a voltage above the coercive field, which would decrease the linearity of the PSC increasing rate and the SW window, and cause the fatigue in ferroelectric material resulting in device breakdown. Also, we used the triboelectric-capacitive coupling effect by touching the polyimide substrate to generating of SW on AiS-TSO, and the measurement of synaptic characteristics of Fe-OFET was conducted as electrical demonstration of AiS-TSO. We could not generate triboelectric voltage output above coercive field by touching. So, we determined that using -10 V magnitude of $V_{rec}$ is appropriate to demonstrate the generation of the synaptic weight originated from ferroelectric dielectric layer. Furthermore, we focus on the fact that our sensory organs have synaptic-like functions that can be used for sensory memory before they are processed in the brain, which are not intended to implement semi-permanent memory. Therefore, we believe that using -10 V of $V_{rec}$ in the minor loop region is enough to demonstrate synaptic function by mimicking the Merkel cell neurite complex. LTP in synapse is very broad[35–37], ranging from minutes to decades, and we can control the STP (seconds) and LTP (hours) of this device by controlling the duration, number and frequency of stimuli.

**Supplementary Note 7**

I **Supplementary Figure 9**, we compared PSC values for the device with poled ferroelectric gate dielectric layer. We conducted poling process by applying the bias of -30 V between gate electrode and drain electrode for 30 min. The channel layer acts as a poling electrode. As shown in **Supplementary Figure 9a**, output characteristics showed increased drain current without saturation, which is different from saturation behavior of unpoled device. No saturation in the poled device is attributed to internal field generated by remnant polarization of gate insulating layer. This result corresponds to our previous investigation about poled Fe-OFET.[5,38] The generated internal field in the poled device acts as negative bias, which enhances accumulation of holes in p-type organic semiconductor channel (**Supplementary Figure 9b**). PSC change and synaptic properties with $V_{rec}$ pulsing were much different for the unpoled and poled device. During pulsing of -10 V $V_{rec}$ is applied to the poled device, the change of PSC is negligibly small since the dipoles in the ferroelectric layer are already aligned (**Supplementary Figure 9c**). So, the dipoles are difficult to be switched further in the same direction with negative $V_{rec}$ pulsing since they are already fully switched by gate biasing of -30 V. Therefore, the synaptic weight of the poled device under negative $V_{rec}$ pulsing is negligible because the polarization was already saturated by poling process. During positive $V_{rec}$ pulsing, the change of PSC in poled device is much smaller than that in unpoled device. The positive $V_{rec}$ pulsing will try to rotate dipoles in the opposite direction to the poled direction of the poled ferroelectric layer resulting in a slight decrease in the PSC because partial switching of dipoles can be more difficult by applying the field opposite to aligned direction of dipoles compared to switching of randomly oriented dipoles (**Supplementary Figure 9d**). Under positive $V_{rec}$ pulsing of the poled device, therefore, a smaller synaptic weight value than that of the unpoled device was also observed. In our approach, partial polarization in ferroelectric gate insulating layer in the unpoled device is used for generating synaptic weight by applying negative or positive $V_{rec}$ pulses. The partial polarization behavior is closely related to mechanism of ferroelectric memory devices.[4,23–27] Therefore, we conclude that the poling process was disadvantageous in generating synaptic weights with potentiation and depression. In our work, the unpoled devices were used for all other measurements to utilize the change in partial polarization as synaptic plasticity.

## Supplementary Note 8

When the device was touched with a bare hand, gloved hand, aluminum foil, stainless steel foil, or label tape paper, the PSC increased due to the electron affinity of polyimide is higher than that of these materials. This is because triboelectric charges (electrons) accumulated on the polyimide, which generated a negative potential on the polyimide and, in turn, an excitatory PSC[44–47]. When the device was touched with PEN or PVC film, triboelectric electrons moved from the polyimide to the touching materials, generating a positive potential on the polyimide and, in turn, an inhibitory PSC[44–47]. Furthermore, when we touched the device by polyimide film, the device response and change of PSC were very small because the triboelectric effect was the smallest (**Supplementary Figure 15**). This phenomenon indicates that triboelectric-capacitive coupling is the dominant mechanism in AiS-TSO.

## Supplementary Note 9

For further investigation about the mechanism of our AiS-TSO, we characterized the response of PSC depending on touching object, temperature and bending strain. First, we touched the AiS-TSO with polyimide (PI) film and finger varying the temperature. As shown in **Supplementary Figure 17a**, the PSC change ($\Delta I_{PSC}/ I_{PSC,i}$)of AiS-TSO to finger touch was much larger by three orders of magnitude than that to touching with PI at the same temperature (~27 $^{o}$C). In both cases, the $\Delta I_{PSC}/ I_{PSC,i}$ was increased with the temperature increased. These results indicate that the main mechanism of AiS-TSO is triboelectric-capacitive coupling effect even though there is a slight change in the $\Delta I_{PSC}/ I_{PSC,i}$ with the temperature increased.

For further investigation effect of temperature change on the response of PSC, we fabricated the OFET device using PVP (polyvinylpyrrolidone) as gate dielectric layer which has no pyroelectricity compared its response to the AiS-TSO. As shown in **Supplementary Figure 17b**, when we touch the both devices by finger using BT NP(20wt%)/P(VDF-TrFE) and PVP gate dielectrics, we could observe the increase of PSC ($I_{PSC}$) in both devices with the temperature increased. From those results, it can be

confirmed that the response of AiS-TSO is not originated from pyroelectricity. Since the OFET with PVP gate dielectric shows an increase in the $I_{PSC}$ with the temperature increased, an increase in $I_{PSC}$ may be attributed to increase in channel conductance due to thermal generation of carriers. Furthermore, pyroelectric effect is expected to be negligible because we didn't carry out any poling process for generating pyroelectricity. From those data, we could confirm that the pyroelectric effect in AiS-TSO is negligible compared to triboelectric-capacitive effect which is the main mechanism of touch response.

In order to investigate the response of AiS-TSO with BT NP(20 wt%)/P(VDF-TrFE) gate dielectric to mechanical strain, we also measured $\Delta I_{PSC}/ I_{PSC,i}$ of the device to tensile and compressive bending strains and compared to that to finger touch. As shown in **Supplementary Figure 17c**, when the AiS-TSO is touched by finger and bent with the bending radius of 2 mm for around 10 s, The $\Delta I_{PSC}/ I_{PSC,i}$ under finger touch was much larger compared to that under bending. Since the bending strain does not induce triboelectric-capacitive effect, only a small response was observed with no synaptic weight. Observed increase and decrease in $\Delta I_{PSC}/ I_{PSC,i}$ of the AiS-TSO may be attributed to piezoresistive effect in the pentacene channel due to increase and decrease of hole carriers in the semiconductor channel under compressive and tensile bending strain, respectively. With a small change in PSC under bending, the device could not generate synaptic weight. These results also indicate that main mechanism of synaptic weight generation is triboelectric-capacitive coupling effect.

**Supplementary Note 10**

Firstly, working principle of POSFET is much different from that of AiS-TSO. POSFET touch sensor utilizes the piezoelectric response of the piezoelectric gate dielectric while AiS-TSO does not utilize the piezoelectric effect of the gate dielectric. In POSFET, touch stimuli induce i) the displacement of polarized piezoelectric material and ii) change the electric field in piezoelectric material, which modulates the carrier density in the channel and, in turn, the drain current (**Supplementary Figure 18a**). Here, the piezoelectric layer upon pressurizing induces change in dipole alignment resulting in change in effective gate electric field[49–51]. Therefore, POSFET needs intended poling process for generating the saturated

remnant polarization, $P_r$, as much as possible to generate piezoelectric voltage enough to modulate the drain current. In AiS-TSO, on the other hand, i) triboelectrification between skin and polyimide substrate (described as receptive part in the manuscript) generates triboelectric charges and ii) coupled capacitive effect in the receptive part induces the partial dipole switching in ferroelectric material and, in turn, change in the drain current (**Supplementary Figure 18b**). Therefore, mechanisms of generating and transduction of energy are different, in which the POSFET sensor uses piezoelectric effect while our AiS-TSO uses triboelectric-capacitive coupling effect between skin and receptive part. Those mechanisms have studied by theoretical analyses on triboelectric effect[45,52–54] or tribotronics[45,55,56] although AiS-TSO has uniqueness in intrinsic-synaptic function and structure mimicking MCNCs.

Secondly, the functions of sensor have differences. Both sensors have a common function with energy transducer from mechanical to electrical as a device of mimicking mechanoreceptors, but AiS-TSO adds intrinsic synaptic functions and enhances the functionalities for mimicking mechanoreceptors (mimicking synaptic functions of Merkel cell neurite complex). Differently from POSFET, we do not carry out intended poling process to generate $P_r$ in ferroelectric material. Instead, we induce modulation of polarization with touch stimuli causing dipole switching depending on nature of stimuli. Therefore, due to the characteristics of ferroelectric material, the conductivity changes in the channel changes are inherently endowed with information of touch stimuli. In conclusion, we could induce SW through the modulation of polarization switching in ferroelectric layer under varying stimuli of touch resulting in inherent change in post-synaptic current and, in turn, modulation of SW. Thus, AiS-TSO has the advantages of a simple structure and manufacturing process, and unlike other mechanoreceptor-mimetic sensors whose only detection function has been reported, AiS-TSO has an intrinsic synaptic function that mimics the Merkel cell neurite complex.

## Supplementary References


1. Kim, D., Hwang, B., Tien, N. T., Kim, I. & Lee, N. Effects of piezoresistivity of pentacene channel in organic thin film transistors under mechanical bending. *Electron. Mater. Lett.* **8**, 11–16 (2012).
2. Kim, Y. *et al.* Flexible textile-based organic transistors using Graphene / Ag nanoparticle electrode. *Nanomaterials* **6,** e147 (2016).
3. Ding, Y. *et al.* Flexible small-channel thin-film transistors by electrohydrodynamic lithography. *Nanoscale* **9**, 19050-19057 (2017).
4. Liu, C., Fujimoto, Y. & Tanaka, Y. Flexible impact force sensor. *Journal of Sensor Technology* **4**, 66–80 (2014).
5. Tien, N. T., Trung, T. Q., Seoul, Y. G., Kim, D. Il & Lee, N. Physically responsive field-effect transistors with giant electromechanical coupling induced by nanocomposite gate dielectrics. *ACS Nano* **5**, 7069–7076 (2011).
6. Yurchuk, E. *et al.* $HfO_2$ based Ferroelectric field-effect transistors with 260 nm channel length and long data retention. *4th IEEE International Memory Workshop* (2012)
7. Yurchuk, E. *et al.* Impact of scaling on the performance of $HfO_2$ based ferroelectric field effect transistors. *IEEE Transactions on Electron Devices*. **61**, 3699–3706 (2014).
8. Muller, J. *et al.* Nanosecond polarization switching and long retention in a novel MFIS-FET Based on Ferroelectric $HfO_2$. *IEEE Electron Device Letters*. **33**, 185–187 (2012).
9. Xiao-Wen,J. *et al.* $MoS_2$ field effect transistors with Lead Zirconate Titanate ferroelectric gating. *IEEE Electron Device Letters*. **36**, 784–786 (2015).
10. Park, B.E. *et al.* Practical characteristics of organic ferroelectric-gate FETs: Ferroelectric-gate field effect transistors with flexible substrates, *Ferroelectric- gate field effect transistor memories* **131**, 227-262 (2016).
11. Unni, K. N. N., Bettignies, R., Seignon, S.D. & Nunzi, J.M. A nonvolatile memory element based on an organic field-effect transistor. *Appl.Phys.Lett.* **85**, 1823–1825 (2004).
12. Sugano, R. *et al.* Switching time in ferroelectric organic field-effect transistors. *Adv.Sci.* **215,** 1701059 (2018).
13. Nguyen, V., Zhu, R. & Yang, R. Environmental effects on nanogenerators. *Nano Energy* **14**, 49-61 (2014).
14. Nguyen, V. & Yang, R. Effect of humidity and pressure on the triboelectric nanogenerator. *Nano Energy* **2**, 604–608 (2013).
15. Gottschaldt.K.M & Vahle-Hinz. C. Merkel cell receptors : structure and transducer function. *Science* **214**, 183–186 (1981).
16. Maksimovic, S., Baba, Y. & Lumpkin, E. A. Neurotransmitters and synaptic components in the Merkel cell-neurite complex, a gentle-touch receptor. *Ann. N. Y. Acad. Sci.* **1279**, 13–21 (2013).
17. Woo, S., Lumpkin, E. A. & Patapoutian, A. Merkel cells and neurons keep in touch. *Trends Cell Biol.***25**, 74–81 (2015).
18. Ikeda, R. *et al.* Merkel cells transduce and encode tactile stimuli to drive Ab-afferent impulses. *Cell* **157**, 664–675 (2014).
19. Hao, J., Bonnet, C., Amsalem, M., Ruel, J. & Delmas, P. Transduction and encoding sensory information by skin mechanoreceptors. *Pflugers Arch.* **467**, 109–119 (2015).

20. Tello, B. M., Chiesa, M., Duffy, C. M. & Sirringhaus, H. Charge trapping in intergrain regions of pentacene thin film transistors, *Adv.Funt.Mater.* **18**. 3907–3913 (2008).

21. Ha, R. & Batlogg, B. Gate bias stress in pentacene field-effect-transistors : Charge trapping in the dielectric or semiconductor. *Appl.Phys.Lett.* **99**, 083303 (2011).

22. Fujisaki, S., Ishiwara, H. & Fujisaki, Y. Low-voltage operation of ferroelectric poly(vinylidene fluoridetrifluoroethylene) copolymer capacitors and metal-ferroelectric-insulator-semiconductor diodes. *Appl. Phys. Lett.* **90**, 162901 (2007).

23. Kim, E. J., Kim, K. A. & Yoon, S. M. Investigation of the ferroelectric switching behavior of P(VDF-TrFE)-PMMA blended films for synaptic device applications. *J. Phys. D. Appl. Phys.***49**, 075105 (2016).

24. Hu, W.J. *et al.* Universal ferroelectric switching dynamics of vinylidene fluoride-trifluoroethylene copolymer films. *Sci. Rep.* **4**, 4772 (2014).

25. Yoon, S. *et al.* Nonvolatile memory thin-film transistors using an organic ferroelectric gate insulator and an oxide semiconducting. *Semicond. Sci. Technol.* **26,** 4869-4874 (2011).

26. Valiyaneerilakkal, U. & Varghese, S. Poly (vinylidene fluoride-trifluoroethylene )/barium titanate nanocomposite for ferroelectric nonvolatile memory devices. *AIP. Adv.* **3,** 11–16 (2014).

27. Engel, S., Smykalla, D., Ploss, B. & Gräf, S. Effect of ( Cd : Zn ) S Particle concentration and photoexcitation on the electrical and ferroelectric properties of ( Cd : Zn ) S / P ( VDF-TrFE ) composite films. *Polymers* **9**, e650 (2017).

28. Xu, T., Xiang, L., Xu, M., Xie, W. & Wang, W. Excellent low-voltage operating flexible ferroelectric organic transistor nonvolatile memory with a sandwiching ultrathin ferroelectric film. *Sci. Rep.***7**, 1–7 (2017).

29. Yoon, S., Kim, E., Kim, Y. & Ishiwara, H. Adaptive-learning functions of ferroelectric field-effect transistors for synaptic device applications. *2017 International Symposium on Nonlinear Theory and Its Applications*, 314-317 (2017).

30. Oh, S., Kim, T., Kwak, M., Song, J. & Woo, J. HfZrO x -Based ferroelectric synapse device with 32 levels of conductance states for neuromorphic applications. *IEEE Electron Device Lett.* **38**, 732–735 (2017).

31. Lee, D. *et al.* Multilevel data storage memory using deterministic polarization control. *Adv. Mater.* **24**, 402–406 (2012).

32. Zhao, D. *et al.*Retention of intermediate polarization states in ferroelectric materials enabling memories for multi-bit data storage. *Appl. Phys. Lett.* **108,** 232907, (2016).

33. Lee, K. *et al.* Stable subloop behavior in ferroelectric Si-doped $HfO_2$. *ACS Appl. Mater. Interfaces* **11**, 38929–38936 (2019).

34. Kyunys, B., Lurchuk, V., Meny, C., Majjad, H., & Doudin, B. Sub-coercive and multi-level ferroelastic remnant states with resistive readout. *Appl. Phys. Lett.* **104**, 232905 (2014).

35. Zucker, R. S. & Regehr, W. G. Short-term synaptic plasticity. *Annu. Rev. Physiol.* **64**, 355–405 (2002).

36. Schall, J. D. Neural basis of deciding, choosing and acting. *Nature Rev. Neurosci.* **2**, 33-42 (2001).

37. Mcgaugh, J. L. Memory — a Century of Consolidation. *Science* **287**, 248-251 (2000).

38. Tien, N. T. *et al.* A flexible bimodal sensor array for simultaneous sensing of pressure and temperature. *Adv. Mater.* **26**, 796–804 (2014).

39. Zeng, B. *et al.* 2-bit/cell operation of $Hf_{0.5}Zr_{0.5}O_2$ based fefet memory devices for NAND applications.

*IEEE J. Electron Devices Soc.* **7**, 551–556 (2019).

40. Tomas, J., Bellaiche, L. & Bibes, M. Learning through ferroelectric domain dynamics in solid-state synapses. *Nat.Commun.* **8**, 14736 (2017).

41. Kim, M. K. & Lee, J. S. Ferroelectric analog synaptic transistors. *Nano Lett.* **19**, 2044–2050 (2019).

42. Jerry, M., Dutta, S., Kazemi, A., Ni, K. & Zhang, J. A ferroelectric field effect transistor based synaptic weight cell. *J. Phys. D. Appl. Phys.* **51**, 433001 (2018).

43. Duiker, H. M. *et al.* Fatigue and switching in ferroelectric memories : Theory and experiment fatigue and switching in ferroelectric memories *J. Appl. Phys.* **68**, 5783 (1990).

44. Zou, H. *et al.* Quantifying the triboelectric series. *Nat. Commun.* **10**, 1427 (2019).

45. Hu, W., Zhang, C. & Wang, Z.L. Recent progress in piezotronics and tribotronics. *Nanotechnology* **30**, 042001 (2019).

46. Yang, Y. *et al.* Human skin based triboelectric nanogenerators for harvesting biomechanical energy and as self-powered active tactile sensor system. *ACS Nano* **7**, 9213–9222 (2013).

47. Yu, A., Zhu, Y., Wang, W. & Zhai, J. Progress in triboelectric materials : toward high performance and widespread applications. *Adv. Funct. Mater.* **29,** 1900098 (2019).

48. Cho, S. W., Kim, D. Il, Hwang, B. U. & Lee, N. Stability of pentacene thin film transistors under simultaneous mechanical bending and heating. *J. Nanosci.***13**, 8002–8006 (2013).

49. Dahiya, R. S., Lorenzelli, L., Metta, G. & Valle, M. POSFET devices based tactile sensing arrays. *Proc. 2010 IEEE Int. Symp. Circuits Syst.* 893–896 (2010).

50. Adami, A., Dahiya, R. S., Collini, C., Cattin, D. & Lorenzelli, L. POSFET touch sensor with CMOS integrated signal conditioning electronics. *Sensors Actuators A. Phys.***188**, 75–81 (2012).

51. Dahiya, R. S., Adami, A., Collini, C. & Lorenzelli, L. POSFET tactile sensing arrays using CMOS technology. *Sensors Actuators A. Phys.* **202**, 226–232 (2013).

52. Zhang, C. *et al.* Contact electrification field-effect transistor. *ACS Nano.* **8**, 8702–8709 (2014).

53. Shankaregowda, S. A. *et al.* Nano energy single-electrode triboelectric nanogenerator based on economical graphite coated paper for harvesting waste environmental energy. *Nano Energy* **66**, 104141 (2019).

54. Niu, S. & Wang, Z. L. Theoretical systems of triboelectric nanogenerators. *Nano Energy* **14**, 161–192 (2014).

55. Gao, G. *et al.* Triboiontronic transistor of $MoS_2$. *Adv. Mater.* **31**, 1–10 (2019).

56. Zhang, C. & Wang, Z. L. Tribotronics—A new field by coupling triboelectricity and semiconductor. *Nano Today* **11**, 521–536 (2016).
